# A framework for the general design and computation of hybrid neural networks

**DOI:** 10.1038/s41467-022-30964-7

**Published:** 2022-06-14

**Authors:** Rong Zhao, Zheyu Yang, Hao Zheng, Yujie Wu, Faqiang Liu, Zhenzhi Wu, Lukai Li, Feng Chen, Seng Song, Jun Zhu, Wenli Zhang, Haoyu Huang, Mingkun Xu, Kaifeng Sheng, Qianbo Yin, Jing Pei, Guoqi Li, Youhui Zhang, Mingguo Zhao, Luping Shi

**Affiliations:** 1 Center for Brain-Inspired Computing Research (CBICR), Beijing Advanced Innovation Center for Integrated Circuits, Optical Memory National Engineering Research Center, & Department of Precision Instrument, Tsinghua University, 100084 Beijing, China; 2 IDG/McGovern Institute for Brain Research at Tsinghua University, 100084 Beijing, China; 3 Lynxi Technologies Co., Ltd, 100080 Beijing, China; 4 Department of Automation, Tsinghua University, 100084 Beijing, China; 5 Department of Biomedical Engineering, Tsinghua University, 100084 Beijing, China; 6 Department of Computer Science and Technology, Tsinghua University, 100084 Beijing, China

**Keywords:** Electrical and electronic engineering, Information technology

## Abstract

There is a growing trend to design hybrid neural networks (HNNs) by combining spiking neural networks and artificial neural networks to leverage the strengths of both. Here, we propose a framework for general design and computation of HNNs by introducing hybrid units (HUs) as a linkage interface. The framework not only integrates key features of these computing paradigms but also decouples them to improve flexibility and efficiency. HUs are designable and learnable to promote transmission and modulation of hybrid information flows in HNNs. Through three cases, we demonstrate that the framework can facilitate hybrid model design. The hybrid sensing network implements multi-pathway sensing, achieving high tracking accuracy and energy efficiency. The hybrid modulation network implements hierarchical information abstraction, enabling meta-continual learning of multiple tasks. The hybrid reasoning network performs multimodal reasoning in an interpretable, robust and parallel manner. This study advances cross-paradigm modeling for a broad range of intelligent tasks.

## Abstract (web summary)

Hybrid neural networks combine advantages of spiking and artificial neural networks in the context of computing and biological motivation. The authors propose a design framework with hybrid units for improved flexibility and efficiency of hybrid neural networks, and modulation of hybrid information flows.

## Introduction

Different from task-specific narrow artificial intelligence, artificial general intelligence (AGI) with the characteristics of human intelligence is expected to excel in scenarios that lack the following conditions: sufficient data, clearly defined problems, complete knowledge, static states, and a single system. The incorporation of computer-science-oriented and neuroscience-oriented computing approaches is widely regarded as a promising direction in the development of AGI^1–5^. Spiking neural networks (SNNs) and artificial neural networks (ANNs) are the representative models of these two approaches, and each has unique advantages. Hence, there is a growing trend of merging these models to leverage their advantages^6–13^. However, the radical differences between SNNs and ANNs^8,10,11,14–16^, such as coding schemes, synchronization methods, and neuronal dynamics, pose great challenges for merging. Recently, a cross-paradigm hybrid neuromorphic computing hardware platform was developed to support a wide range of ANN and SNN models^6^. A growing number of research teams are adopting various features to better support different networks in their neuromorphic designs. For example, Intel, IBM, and the University of Manchester recently undertook hybrid designs in their Loihi^17^, In-memory computing^11^, and Spinnaker^18^, respectively. Meanwhile, a unified system hierarchy with neuromorphic completeness^7^ for brain-inspired computing has also been developed, providing general support for executing different types of programs and network models on various typical types of hardware^7^. Collectively, these provide powerful hardware platforms and software deployment tools for the development of hybrid neural networks (HNNs). On the other hand, there are also some attempts to combine SNNs and ANNs to build hybrid models from different perspectives, such as information processing^8,9^, computational efficiency^10^, or establishing models that incorporate more biological attributes^11,12^. However, they narrowly focus on using certain features of ANNs and SNNs to solve specific tasks. A general framework for versatile tasks that can take full advantages of both models is essential but still lacking.

In this study, we propose a framework to support the general design and computation of HNNs at multiple scales and multiple domains by decoupling ANNs and SNNs models and using hybrid units (HUs) as their linkage interfaces. In particular, we consider that, unlike the homogenous information in pure SNNs or ANNs, the hybrid information flows in HNNs have heterogeneity at different spatial and temporal scales. If SNNs and ANNs were directly coupled neuron-to-neuron, HNN models would be inefficient and unmanageable when their architecture becomes complex. With the support of HUs, our decoupled approach not only inherits the key characteristics of SNNs and ANNs, but also provides greater flexibility in the design of hybrid models, thereby making their strengths and techniques more self-contained. An HU is an information transformation model with intermediate representations so that it can bridge the gap between SNNs and ANNs, whose parameter configurations can be designed according to domain knowledge or learned to adapt. Under mild conditions, HUs would enable the development of HNNs with general-purpose computation. This framework provides a high degree of freedom for building interwoven hybrid network models, and an intrinsic capability to process rich spatiotemporal information in a hierarchical and multi-domain manner^6^.

## Results

### The framework of hybrid neural networks

**Fig. 1:**
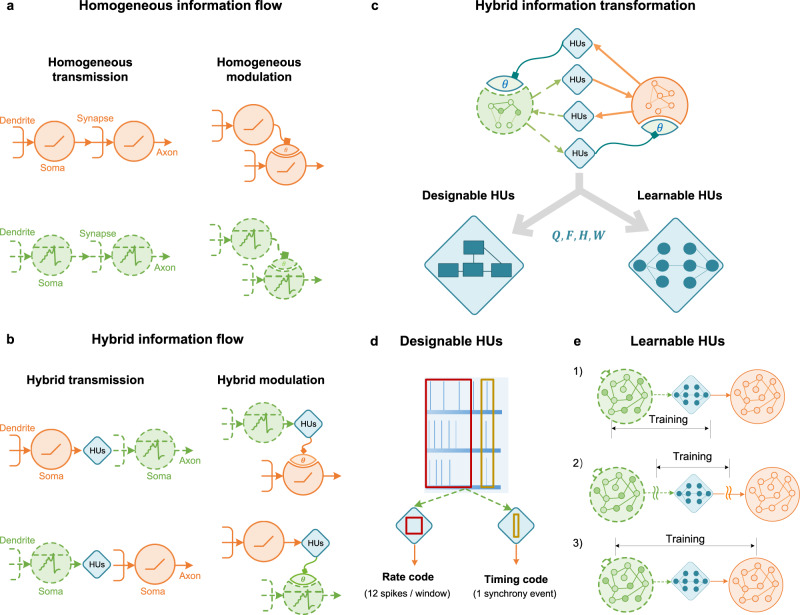
Illustration of the proposed HNN framework. **a** Homogeneous information flow includes synchronous-continuous signals (solid orange lines) of ANN neurons (orange circle) and asynchronous-discrete signals (dashed green lines) of SNN neurons (green circle), respectively. Each case includes direct transmission (left, sharp arrow head) and indirect modulation (right, square arrow head pointing to parameters θ of modulated neurons). **b** Hybrid information flow is transformed by HUs (blue squares). **c** HUs have several basic computation steps, including truncating (W(t)), filtering (H(t)), non-linearity (F), and discretization (Q). These operations can be achieved by knowledge-driven manual design or data-driven automatic learning. **d** Designable HUs are configured according to the target coding schemes by prior knowledge and predefined mapping (The long time-scale red window for rate coding, short time-scale yellow window for timing code (e.g., synchrony)). **e** Learnable HUs can be configured in three learning ways: (1) jointly training with frontend/backend networks, (2) independent training, and (3) training with complete models.

Figure 1 illustrates the core components and key characteristics of the proposed framework, including hybrid information flows and HUs. In addition to the homogenous information in pure SNNs or ANNs (Fig. 1a), there are two basic types of hybrid information flow in HNNs: hybrid transmission and hybrid modulation (Fig. 1b). Hybrid transmission directly affects neuron states through synaptic transmission between heterogeneous networks, whereas hybrid modulation largely exerts an indirect influence on neuron states by adjusting parameters of neurons or synapses, such as neuronal thresholds and synaptic weights. These hybrid information flows have different spatiotemporal scales, which provide rich coding schemes and flexible configurations of hybrid networks. The interaction between these factors further gives rise to a wide diversity of hybrid models but also poses great challenges to the transformation of hybrid information.

To bridge the synchronous real-valued representation of ANNs and the asynchronous spike representation of SNNs, HUs with generalized asynchronous real-valued intermediate representation are adopted (Fig. 1c, also see Methods). The HUs coordinate synchronization, time-scale, and coding schemes to address the hybrid information transformation in general. The premise of information transformation is to determine the input and output domains. In the HU model, the raw information *X* can be truncated by a window function *W* to synchronize the time scales between the input and output information. Then, the spatiotemporal information can be extracted from the input signals by using the kernel function *H* and nonlinear operation *F* to achieve the domain transformation. Finally, an optional signal operation *Q*, such as thresholding or discretization, can be used to support the diverse representation characteristics of the target domain. Collectively, the output of HUs can be formalized as

1 $$Y={{{{{\rm{HU}}}}}}[{{{{{\rm{X}}}}}}]=Q\cdot F\cdot H\cdot W(X).$$

Unlike conventional signal converters that perform direct or predefined conversions, HUs are reconfigurable. Their key components, such as *H* and *F*, can be parameterized, facilitating flexible conversion strategies to meet the various requirements of diverse coding schemes. The two operations, *H* and *F*, ensure the universal approximation capability of HUs for arbitrary approximation (see Supplementary Material).

We provide two methods to configure the parameters of HUs: manual design and automatic learning (Fig. 1c–e). When the relationship between heterogeneous representations is deterministic, simple, and known, it is convenient to configure a designable HU with prior knowledge (Fig. 1d). However, in most cases, the relationship is non-deterministic, complex, or unknown, in which a learnable HU is more desirable. For non-deterministic cases, due to the lack of information in the frontend networks, it is difficult to fully satisfy the requirements of the backend networks. Learnable HUs can use empirical learning, e.g., naïve Bayes classifier for probabilistic mapping, to approximately meet the requirements. For complex cases, where the relationship may be deterministic, but the transformation is too complex to be programmed, learnable HUs can save the effort. For unknown cases, where the relationship may be deterministic and simple, but lack prior knowledge to accomplish a design, learnable HUs may automatically identify the principle behind empirical data, e.g., Kepler’s laws. We propose three possible learning approaches for learnable HUs (Fig. 1e): (1) installed in the frontend or backend network and jointly trained with the connected networks; (2) modeled separately by setting independent optimization goals; and (3) trained with the complete model. Collectively, both accurate and approximate models can be established using designable and learnable HUs.

AGI systems are expected to possess the intrinsic capabilities to process rich spatiotemporal information, support vast and complex neural networks in a hierarchical and multi-domain manner, and realize the intertwined cooperation of multiple neural networks^6^. Powered by HUs, HNNs with different network architectures can be built to best meet these needs and handle different applications. We investigate the capabilities of the framework in facilitating the design of hybrid models using three experiments: (1) a hybrid sensing network (HSN) with multi-pathway hybrid transmissions realizes multi-pathway sensing; (2) a hybrid modulation network (HMN) with hierarchical information abstraction accomplishes meta-continual learning (MCL) of multiple tasks; and (3) a hybrid reasoning network (HRN) integrating multimodal and multi-domain information performs logical reasoning in an interpretable, robust and parallel manner.

### Hybrid sensing network

**Fig. 2:**
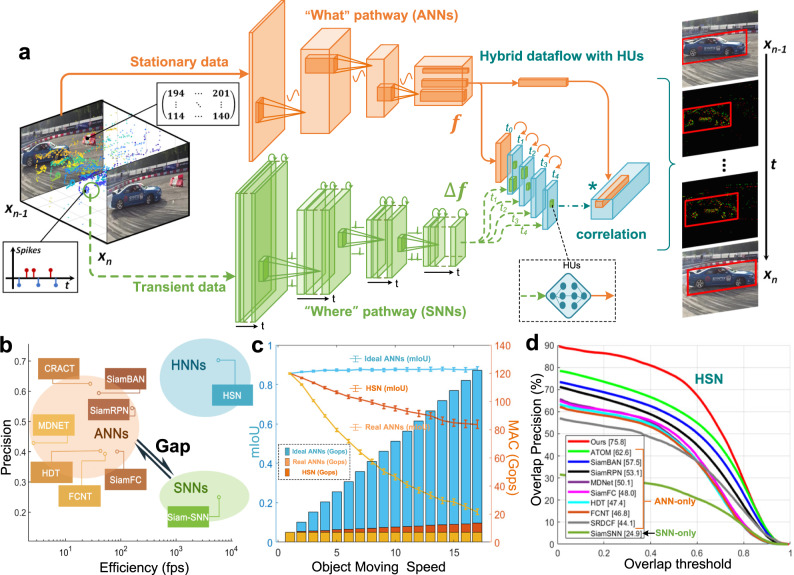
Multi-pathway HSN for high-speed vision perception. **a** The architecture of HSN. The orange part represents the “what” pathway processing the static information from APS, whereas the green part represents the “where” pathway processing the dynamic information from DVS. The “***f***” and “Δ***f***” indicates the feature with orange and “Δfeature” with green, respectively. **b** Precision and speed comparison of ANNs, SNNs, and HNNs. **c** Network performance for different object-moving speeds and computational cost of ideal ANNs, real ANNs, and HSNs on CLEVRER-DAVIS datasets (see Supplementary Material). The *x*-axis refers to the results of object-moving speed accelerated by *x* times. The left side axis is the speed-precision curve and the right-side axis is the speed-MAC bar plot. All the experiments in this demo are independently run four times with the same setting and different initializations. The error bar represents the standard deviation of the mean IoU results. **d** Network performance comparison and success plots of HSN, and the state-of-the-art trackers on NFS-DAVIS and PRED. The data sources for comparison come from the published work, including CRACT^21^, SiamBAN^22^, SiamRPN^23^, MDNET^24^, HDT^51^, SiamFC, FCNT^26^, Siam-SNN^19^, and SRDCF^27^.

We design an HSN with multi-pathway hybrid transmission to demonstrate the advantages of hybrid models in visual perception. It processes visual information using a divide-and-conquer strategy. The HSN first divides information into static and transient signals, then conquers them independently through “what” and “where” pathways in shallow layers, and finally combines the static and dynamic outputs from ANNs and SNNs in deeper layers through learnable HUs (Fig. 2a). The learnable HUs are jointly trained with the frontend ANN and SNN. This enables visual features to be reused and dynamically updated, achieving significant improvements over single-paradigm approaches.

Note that if we adopted a pure-ANN model in this task, it would process each frame independently and recalculate all feature maps entirely, even if consecutive frames are highly redundant. If we adopted a homogeneous SNN model, like SiamSNN^19^, it would be difficult at present to represent high-precision tracking information (i.e., the bounding box) and process complex frame-based information using spiking signals, resulting in poor tracking precision. Alternatively, our HSN divides the features into *f* and Δ*f*, which correspond to the static and dynamic parts (see Methods), producing the final prediction by recalculating the dynamic part, which greatly reduces the computational redundancy through an efficient coding strategy. Therefore, the HSN can leverage the advantages of ANNs and SNNs to simultaneously improve efficiency and precision (Fig. 2b).

We further quantitatively evaluated the tracking performance and computational cost of the HSN with respect to the object’s moving speed. We adopted the widely accepted “streaming accuracy“^20^, which fully considers the real latency in the hardware processing pipeline and the computational resource constraints (Fig. 2c). For comparison, we also assessed a pure-ANN model under ideal conditions without considering bandwidth and computing resource constraints (standard offline evaluation), and under real-world conditions (online test based on Tianjic chips^20^). The ANN achieved a high tracking accuracy of 0.85 mean intersection-over-union (mIoU) in the ideal scenario, whereas its performance dropped dramatically to 0.33 mIoU in the real-world scenario because of the efficiency of ANNs. By contrast, since HSN inherited the event-driven feature of SNNs, it maintained a high tracking accuracy (0.679 mIoU) even in the real-world scenario, which was more than 100% higher than that of the real-ANN. We also compared our model with two types of trackers on NFSDAVIS datasets (Fig. 2d): (1) ANN-based methods (CRACT^21^, SiamBAN^22^, SiamRPN^23^, MDNET^24^, SiamFC^25^, FCNT^26^, and SRDCF^27^), and (2) an SNN-based method (Siam-SNN^19^). Our results indicated that the HSN achieved a high tracking speed (5952 FPS) and a high power efficiency (130 μJ/inference), which were 11 times and 2 times higher than those of pure ANNs, respectively, demonstrating the great potential of HNN in visual perception tasks (*see Methods*).

### Hybrid modulation network

**Fig. 3:**
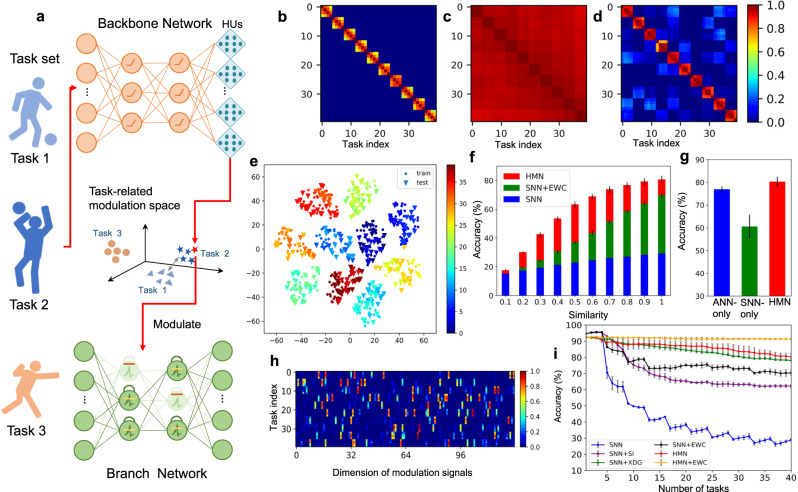
HMN for MCL tasks. **a** Architecture of HMN, consisting of an ANN-based backbone network to extract task-level information and an SNN-based branch network to perform specific tasks. Learnable HUs are adopted for adaptively adjusting modulation signals to meet the requirements of the branch network. The HUs are installed at the backend of the backbone network (Fig. 1e-(1)), and are jointly trained by the task similarity objective. **b** Similarity matrix of tasks, calculated based on the Hamming distance between the permutation indices of tasks in this experiment. **c**, **d** The correlation matrix of mean activations of hidden neurons of the HMN and the single SNN, respectively. **e** T-SNE embeddings of the sample-specific modulation signals generated by the backbone network. The triangle denotes the modulation signal of the test samples of the backbone network. **f** Mean accuracy of different models on unlearned tasks with different levels of similarity. **g** Mean accuracy of the ANN-only model and the SNN-only model with a similar architecture of HMN, respectively. **h** The first 128 dimensions of the task-specific modulation signals before thresholding. **i** Mean accuracy of different models on learned tasks versus the number of learned tasks. All the experiments in this demo are independently run four times with the same setting. The error bar represents the standard deviation of the results.

Deep-learning-based methods typically employ a single end-to-end network structure to solve specific tasks; however, it is difficult to construct multi-network systems. In this section, we use the framework to develop a hierarchical multi-network system with a task-driven parameter modulation mechanism (see Methods), which we call the HMN. Then we investigate the model’s abilities in solving MCL problems. The HMN provides a hierarchical abstraction of the tasks and combines the advantages of different types of networks. Specifically, we construct an ANN-based backbone network to generate continuous signals representing task-related information, and construct an SNN-based branch network with rich dynamics to perform specific tasks. The HUs are installed in the backbone network, and generate modulation signals by adjusting the neuron thresholds to control the neuronal dynamic behaviors of the branch network. The overall HMN workflow is illustrated in Fig. 3a. Unlike most existing context-based modulation methods^28,29^, the HMN explicitly incorporates task similarity to train the backbone network and takes advantage of HNNs to solve MCL problems.

We validated the HMN on the permuted N-MNIST dataset (see Supplementary Material). The sequentially learned tasks were synthesized in a grouped fashion. Tasks within a group are similar, whereas tasks in different groups are dissimilar (Fig. 3b). As illustrated by the t-SNE in Fig. 3e, the modulation signals (Fig. 3h) generated by the HUs for tasks in the same group are clustered together, while those in different groups are separated. This indicates that the HUs generate appropriate modulation signals according to task similarity. As shown in Fig. 3i, with the support of proper modulation signals, the mean accuracy of the branch network after learning 40 tasks exceeds that of single SNNs and SNNs combined with typical continual learning methods, such as context-dependent gating^29^, elastic weight consolidation (EWC)^30^, and synaptic intelligence^31^. The HMN can be further combined with regularization-based methods, such as EWC, to improve performance. The accuracy comparison on unlearned tasks with different levels of similarity indicates that the HMN achieves superior performance on similar unlearned tasks, which can be attributed to the high-level management capabilities of the backbone network (Fig. 3f). It is worth noting that the HMN demonstrates scaling benefits of parameter efficiency on continual learning tasks (see Supplementary Material), which are brought about by hierarchical multi-network architectures and diverse parameter modulations between heterogeneous networks. With the continuous learning of many tasks, the accuracy of single models saturates, but that of the HMN keeps improving. Ablation experiments (Fig. 3g) of the ANN-only model or the SNN-only-model with a similar modulation architecture of the HMN were also conducted (see Supplementary Material). Their inferior accuracies validate the unique benefits of the hybrid approach.

We further quantitatively analyzed the HMN by presenting the correlation matrix of the mean activations of the hidden neurons of the branch network across different tasks after sequential training (Fig. 3d), and compared it with a pure-SNN model (Fig. 3c). It can be noted that the activations of the SNN on different groups of tasks are highly correlated, suggesting that the same parameters are mostly used for uncorrelated tasks, which is the major cause of catastrophic forgetting. By contrast, the branch network in the HMN activates only a part of the same set of neurons to perform similar tasks, while activating other neurons to perform uncorrelated tasks. Hence, the HMN not only avoids parameter interference between uncorrelated tasks but also enhances parameter reuse between correlated tasks, thereby improving the efficiency of MCL problems and avoiding catastrophic forgetting.

### Hybrid reasoning network

Reasoning in complex multimodal dynamic environments is important for AGI development. We consider a visual question answering task to exploit the advantages of the proposed framework in reasoning from a new perspective. We design an HNN-based neural reasoning model, which is called the HRN. The HRN adopts a multi-network hierarchical structure. Its ANN-based frontend networks employ a connectionist method to learn to process multimodal and multi-domain information from the external environment, while its SNN-based backend network uses an abstract symbolic representation for information processing and performs explainable reasoning. This approach provides an interpretable, robust, parallel, and thus efficient solution for dynamic reasoning.

The central reasoning module is built with integrate-and-fire neurons representing scene-related semantic concepts (e.g., red, color, object) or general functional concepts (e.g., inhibition, excitation, copy). The connections indicate that the working memory functions in three stages: recalling prior knowledge from long-term memory by initialization, storing visual information from perception using Hebb rules, and executing reasoning operations on demands from external stimuli. In the storing phase, we use designable HUs to transform static visual information (e.g., a red object), and learnable HUs to transform dynamic information (e.g., two objects collide). This is because the former process easily maps the frontend object-oriented representations into the backend predefined symbols, and the latter is non-deterministic due to the uncertainty of the event time. By constructing a graph-based SNN reasoning module and associating the ANN perception module for multimodal perception, the HRN can effectively store, reuse, and represent logical relationships with high data and memory efficiency while embedding prior knowledge into graph form, thereby enabling one-shot learning.

**Fig. 4:**
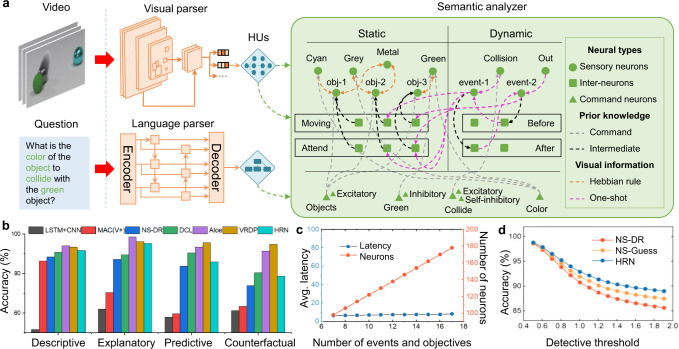
HRN for VQA task. **a** Two ANN-based parsers extract vision and language features, and an SNN-based analyzer executes a two-phase process to construct a graph and reason with it. In the construction phase, the SNN is initialized with prior knowledge, and then the independently trained learnable HUs transmit visual information into spike trains, which activate sensory neurons and intermediate neurons simultaneously in the SNN to embed external information according to the Hebbian rule and one-shot plasticity. In the reasoning phase, designable HUs transmit language information into polychromous activations^52^ activating command neurons to execute the reasoning process. **b** The accuracy of the CLEVRER validation set according to different task types. The reasoning results are compared with CNN-LSTM^35^, MAC(V+)^36^, NS-DR^37^, DCL^38^, Aloe^39^, and VRDP^40^, respectively. **c** The plot of calculation latency and the number of spiking neurons against the increase of target objects. **d** Reasoning robustness to the frontend anomaly data.

We validated an HRN model, with similar frontend feature extractors in NS-DR, on the CLEVRER dataset^32^. In this setting, the HRN abstracts high-level features from images and natural language using a mask RCNN and a sequential generation model (SGM)^33^, respectively. For predictive and counterfactual questions, we further used a propagation network (PropNet)^34^ to process and augment visual features, making the HRN predictive for dynamic motion. The instructions generated by the SGM were converted into spike-based temporal stimuli by designable HUs. The instruction sets were manually set and thus fully understandable. In the reasoning phase, the spiking reasoning module activated the corresponding response nodes, and finally determined the output according to the firing node when the network was in a stable state. Thus, our model achieved accuracies of 91.65%, 95.27%, 85.96%, and 78.81% on descriptive, explanatory, predictive, and counterfactual questions, respectively, compared to other advanced methods^35–37^ (Fig. 4b). We further analyzed the parallelism of the HRN and the reasoning robustness to abnormal commands (Fig. 4c, d). The calculation latency remains nearly unchanged as the number of objects and events increases, indicating the high parallel processing ability of the HRN for an arbitrary number of objects. Another attractive feature of HRN is its robustness to abnormal data processing, which is inevitable in the practical construction of large-scale systems for complex tasks. We examined robustness by gradually relaxing the event detection conditions of the designable HUs. As the detection threshold is relaxed, the number of abnormal data increases (Fig. 4d). This often leads to a type of error in program-based models, resulting in empty outputs (for the NS-DR model) or random guesses (for the NS-Guess model). Conversely, the graph structure of the spiking reasoning network contains prior knowledge that reduces the answering space to a considerably small size. The HRN achieves higher accuracies than the NS-DR and NS-DR-Guess models, which indicates that the HRN is more robust to abnormal data. Besides NS-DR, there are some state-of-the-art models on CLEVRER. DCL^38^ uses a concept learner for efficient training. Aloe^39^ adopts auxiliary self-supervised losses for better object-oriented representations. VRDP^40^ adopts a physical model to achieve accurate physical prediction. In principle, these new techniques are compatible with the HRN, and will help to further improve its model and performance.

It is worth noting that the ANNs and SNNs employed in the above three demonstrations are homogeneous networks, and do not fully exploit features from each other, such as heterogeneous dynamics and connectivity. Incorporating heterogeneous characteristics into homogeneous networks is a potential hybrid approach to further improve the competitiveness of HNNs, which deserves further exploration.

Additionally, we find inspiring analogies between the above demonstrations and biological neural systems. The ANNs and SNNs in HSNs are comparable to the midget-parvocellular (P) pathway and parasol magnocellular (M) pathway in visual information processing, respectively, suggesting that a similar strategy is exploited by the neural system to achieve high-speed and high-precision vision. The backbone network of the HMN has counterparts in the dense neuropeptide network responsible for the slow (minute-scale), diffusive, and fine-grained modulation of cortical fast synaptic transmission. The HRN can be regarded as a highly simplified model of prefrontal working memory, where symbol-like processing needs to be coordinated with sensory grounding extracted from other cortical areas for proper reasoning. Further discussions can be found in Supplementary Material.

## Discussion

Biological neuronal systems embrace multi-scale and multimodal signal communication and information integration. Various coding strategies have been proposed, which suggests that hybrid information transformation is likely to be a requirement for normal cerebral function. Considering these features, we argue that it might be feasible to combine ANNs and SNNs using a general transformation scheme to model “hybrid” properties in neural systems. For instance, mean-field activities, such as fMRI recordings or MEG recordings, can be efficiently modeled using ANNs^3^. Transient activities, such as spike timing synchrony, can be modeled using SNNs. Furthermore, combining different neurons and HUs can enable HNNs to implement various artificial and spiking neuron models, ranging from the classic leaky integrate-and-fire to the complex Hodgkin-Huxley model, which facilitates implementation on neuromorphic computing chips (see Methods). Thus, HNN models can not only be inspired by biological functions but also serve as powerful prototypes to promote future studies on functional neuroscience and practical brain simulations, which can be used to reveal the relationship between a certain human behavior or disease and a certain brain network mechanism^41–43^.

Our proposed framework can greatly exert the strengths of ANNs and SNNs, and facilitate the formation of complementary multi-network models. The abilities of this framework in facilitating HNNs are multifaceted, which we demonstrated experimentally. The HSN appropriately combines the high-precision of ANNs and the high efficiency of SNNs to achieve a record-breaking performance in tracking tasks. The HMN explores the advantages of hybrid modulation in MCL problems, demonstrating the diversity of hybrid information flows for multi-network collaboration. The HRN develops a hybrid architecture to integrate multimodal information and support interpretable logical reasoning in a dynamic environment, demonstrating robustness, high parallelism, and scalability for solving large-scale and complex problems. When encountering complex environments with large uncertainties, learnable HUs can facilitate more adaptive transformation for hybrid representations, leading to superior performance (see Supplementary Material). In summary, the HNN framework can provide flexible and versatile strategies to coordinate heterogeneous networks and exploit their complementary advantages. This paves the way for the development of cross-paradigm network systems for real-world applications and potentially contributes to the development of AGI.

## Methods

### Comparison between artificial neural networks and spiking neural networks

ANNs and SNNs use different approaches to represent, process, and propagate information, thereby exhibiting unique strengths in different scenarios.

In the neuron model, ANNs comprise neurons interconnected with synapses, where the information processing mainly includes a linear input-output transformation and a differentiable, nonlinear activation functions *f*_*a*_^15^. The basic ANN model can be formulized by

2 $${a}^{n}={f}_{a}\left(\sum {w}_{i}{a}_{i}^{n-1}\right),$$

where *w*_*i*_ denotes the weights, *a*^*n*−1^ denotes the activation in *n* − 1 layer. ANNs are primarily used in applications that involve static data. Their models can be extended to include recurrency, thereby leading to so-called recurrent neural networks^11^. By contrast, SNNs comprise neurons interconnected with (dynamic) synapses, where their neural dynamics can represent an input-output transformation involved in versatile neuronal dynamics and a typical non-differentiable threshold-triggered activation *f*_*s*_. The basic SNN model describing membrane potential dynamics *u* can be formalized by,

3 $$\left\{\begin{array}{c}{\tau }_{u}\frac{d{u}^{n}}{{dt}} =-g\left({u}^{n}\left(t\right)\right)+{\sum }_{i}{w}_{i}{s}_{i}^{n-1}\left(t\right)\\ {s}^{n}\left(t\right)={f}_{s}\left({u}^{n}\left(t\right)\right)\end{array}\right.,$$

where *τ*_*u*_ denotes the membrane potential constant, *g*(*u*) denotes the leaky function. *f*_*s*_ is the non-differential spiking function. If the membrane potential *u* exceeds a threshold *v*_th_, *f*_*s*_(*u*) generates a spike *s*^*n*^(*t*) and the membrane potential will be reset.

In signal and coding, ANNs use an analogue and synchronous signal^8,10^ to represent information. They have a unified vectorized distributed coding method and primarily use high-precision activations to propagate information. SNNs use discrete asynchronous spikes^8,10^ to represent information. They have multiple information coding forms (e.g., rate, temporal, burst, and population coding) and use sparse and event-driven spikes to propagate information.

Because of their differences in basic neuron models and signal form, ANNs and SNNs have unique advantages and best-suited scenarios for applications. ANNs can learn nonlinear and complex relationships from large amounts of data. Because of their mature training algorithms, efficient acceleration platform, and suitable dataset, they have demonstrated powerful capability in scenarios that use high-precision and intensive computation^16^. SNNs memorize historical temporal information using intrinsic neuronal dynamics and code information in digital spike trains, thereby enabling event-driven computation. In terms of the data source, their inherent characteristics are suitable for handling event-driven, sparse, or timing data^14^, particularly in neuromorphic sensors^11^, bionic materials^44^, and equipment^8^. In terms of practical implementations, incorporating asynchronous temporal spiking neural dynamics and the physical collocation of neural processing have led to the development of non-von Neumann systems with significantly increased parallelism and reduced energy consumption, demonstrated in chips such as Neurogrid^45^, IBM’s TrueNorth^46^, and Intel’s Loihi^47^.

### Definition and formulation of hybrid units

We formularize HUs as a cascade of computation steps, W, H, F, and Q

4 $$Y={{{{{\rm{HU}}}}}}\left[X\right]=Q\cdot F\cdot H\cdot W\left(X\right)=Q\left[F\left(h\left(t,k\right)\right)\right]$$

where X is the input and *h*(*t*, *k*) is the intermediate representation, which will be introduced below. Y is the m-dimensional output used to feed to the subsequent heterogeneous networks as the inputs or parameters.

**Windowing W**. Since *a*[*k*] and *s*(*t*) are two time series with no intrinsic temporal relation, we need a mechanism to coordinate the time-scale. Thus, X is firstly truncated by a parametric window function $W(t,k,{T}_{s})$, where *T*_*s*_ is a given time window. The result is $X\cdot W(t-k{T}_{s})$.

**Kernel H**. ${{{{{\rm{X}}}}}}\cdot {{{{{\rm{W}}}}}}({{{{{\rm{t}}}}}}-{{{{{\rm{k}}}}}}{T}_{s})$ is then convolved with a kernel function H(t). In this way, the inputs *X* are transformed into an intermediate representation *h*(*t*, *k*). *h*(*t*, *k*) has a compatible format with both *a*[*k*] and *s*(*t*)

5 $$h\left(t,k\right)=\left[X\cdot W\left(t-k{T}_{s}\right)\right]* H\left(t\right).$$

**Nonlinear transformation F and discretization Q**. Based on the intermediate representation, the ultimate transformed results used in transmission or modulation are calculated as follows:

6 $$Y={{{{{\rm{HU}}}}}}\left[X\right]=Q\left[F\left(h\left(t,k\right)\right)\right],$$

where *F* is a nonlinear function used to realize a more complex transformation. *Q* is the discretization operator that can transform continuous signals into spike trains when converting to SNNs or real-valued sequences when converting to ANNs. For example, thresholding in terms of amplitude can discretize a continuous-valued signal to binary spike trains. Evaluating an integral in the time domain is an option to eliminate the continuous-time variable, which converts a continuous-valued signal to a discrete-time sequence. *Q* can be omitted in HUs for hybrid modulation because parameter modulation is not restricted to a signal with a discrete time or value.

*W*, *H*, *F*, *Q* can be parameterized, either manually configured or automatically learned and adapted from the environment. In this manner, Eqs. (5) and (6) provide a flexible conversion strategy for meeting the requirements of various tasks. If necessary, other approaches such as programs or neural networks can also serve as functional parts of the hybrid unit to complete the information conversion.

### Details of the hybrid sensing network

#### The network architecture of the HSN

As shown in Fig. 2a, the HSN consists of a “what” pathway for the static feature *SF*(*t*) extraction, where *SF*(*t*) is the features of the ANNs at time *t*, and a “where” pathway for the dynamic feature Δ*DF*(Δ*t*) prediction, where Δ*DF*(Δ*t*) is the change of features through time during the interval of Δ*t*. Specifically, the ANN and a similar structured SNN process the “what” and “where” information simultaneously and predict the uncaptured frame features $\widehat{{SF}}(t+\triangle t)$ by ${SF}\left(t\right)+{HU}[\triangle {DF}(\triangle t)]$, where the *HU* denotes the learnable HUs transmitting dynamic features of SNNs to ANNs. An object tracking task is then performed by minimizing the distance of the template frame feature *SF*(*t*) and the following predicted features $\widehat{{SF}}(t+\triangle t)$. During the inference phase, the two pathways receive and process different channels of information:

In the “what” pathway, neurons in ANNs communicate with each other using the high-precision and continuous activation by Eq. (2). We provide the ANNs with input image S(*t*) at time *t* and adopt a fully convolutional network as a feature extractor.

In the “where” pathway, we use the linear leaky integrate-and-fire (LIF) model with *g*(*x*) = *x* as described in Eq. (3). To enable spiking dynamics with gradient descent, we adopt an iterative LIF model and use spatiotemporal backpropagation methods^48,49^ that apply the BPTT algorithms to the LIF model as follows,

7 $$\left\{\begin{array}{c}{u}_{t+1}^{n}{{{{{\rm{\& }}}}}}=\left(1-{u}_{t}^{n}\right){e}^{-\frac{{{{{{\rm{d}}}}}}t}{\tau }}{u}_{t}^{n}+{\sum }_{{{{{{\rm{i}}}}}}}{w}_{i}{s}_{t}^{n-1}\\ {s}_{t}^{n}{{{{{\rm{\& }}}}}}=H\left({u}_{t}^{n}-{v}_{{{{{{\rm{th}}}}}}}^{n}\right)\end{array}\right..$$

We use the discrete-time-step to simulate LIF dynamics and use the subscript *t* to distinct different time steps. We use Heaviside function H(u) for the spiking function and take the surrogate function method to approximate its derivative^48–50^.

As shown in Fig. 2a, we input the SNNs with transient information representation in form of spikes $s\left(x,y,\triangle t\right)={\sum }_{{t}_{n}\in \left[t,t+{dt}\right]}\delta (x,y,t-{t}_{n})$ and predict the change of features $\triangle {DF}(\triangle t)={{SF}}^{{\prime} }[s\left(x,y,\triangle t\right)]$, where *SF*′ represents derivative functions of ANNs transformation.

The training process of HSN includes two iterative training phases:

#### Phase 1: the “what” pathway

The “what” pathway of ANNs is trained jointly with gradient backpropagation from three terms of losses: a weighted cross-entropy loss *L*_*c*_ between ground-truth class ${\widetilde{C}}_{k}^{j}$ and predicted classification map ${C}_{k}^{j},j\in \left\{{{{{\mathrm{0,1}}}}}\right\}$, *j* denotes the foreground or background in the *k*^th^ anchor; a smoothed-*L*_1_ loss *L*_1_ between the predicted regression map ${R}_{k}^{j}$ and ground-truth regression map ${\widetilde{R}}_{k}^{j},i\in \left\{{{{{\mathrm{0,1,2,3}}}}}\right\}$, *i* identifies one of the four regression values; and a regularization loss *L*_*R*_ on features

8 $$L={L}_{c}\left({\left\{{C}_{k}^{j},{\widetilde{C}}_{k}^{j}\right\}}_{j,k}\right)+{L}_{1}\left({\left\{{R}_{k}^{i},{\widetilde{R}}_{k}^{i}\right\}}_{i,k}\right)+{L}_{R}.$$

The *L*_*c*_ is defined as:

9 $${L}_{c}=\frac{\mathop{\sum}\limits_{k}w\left({\widetilde{C}}_{k}^{j}\right)\left.\left(-C{p}_{k}^{j},|,{\left\{i={\widetilde{C}}_{k}^{j}\right\}}^{+{ln}({\Sigma }_{i}{e}^{{C}_{k}^{j}})}\right)\right|}{{\Sigma }_{k}{{{{{\rm{w}}}}}}\left({\widetilde{C}}_{k}\right)},$$

where an IoU metric between anchors and the ground-truth target bounding box is used to determine the ground-truth class of each anchor. ${\widetilde{C}}_{k}^{j}\in \{{{{{\mathrm{0,1}}}}},-1\}$ is set to 1 for anchors with IoU larger than 0.45, 0 for anchors with IoU less than 0.2 and −1 otherwise. The anchors with ${\widetilde{C}}_{k}^{j}=-1$ are omitted from the summation. To balance positive and negative samples, loss from each class is weighted by

10 $$w\left({{{{{{\mathrm{class}}}}}}}\right)=\left\{\begin{array}{c}1,{{{{{{\mathrm{class}}}}}}}{{{{{\rm{\& }}}}}}=0\\ 10,{{{{{{\mathrm{class}}}}}}}{{{{{\rm{\& }}}}}}=1\end{array}\right..$$

The *L*_1_ is defined as:

11 $${L}_{1}=\frac{{\sum }_{k\in \{k{{{{{\rm{|}}}}}}{\widetilde{C}}_{k}^{j}=1\}}{\sum }_{i}z({R}_{k}^{i},{\widetilde{R}}_{k}^{i})}{{N}_{k}{N}_{i}},$$

12 $$z\left(a,b\right)=\left\{\begin{array}{c}0.5{\left(a-b\right)}^{2},\left|a-b\right| < 1\\ \left|a-b\right|-0.5,\left|a-b\right|\ge 1\end{array}\right..$$

For an anchor $\left[{{{{{{\rm{x}}}}}}}_{{{{{{\rm{a}}}}}}},{{{{{{\rm{y}}}}}}}_{{{{{{\rm{a}}}}}}},{{{{{{\rm{w}}}}}}}_{{{{{{\rm{a}}}}}}},{{{{{{\rm{h}}}}}}}_{{{{{{\rm{a}}}}}}}\right]$ and a target bounding box $\left[{{{{{{\rm{x}}}}}}}_{{{{{{\rm{t}}}}}}},{{{{{{\rm{y}}}}}}}_{{{{{{\rm{t}}}}}}},{{{{{{\rm{w}}}}}}}_{{{{{{\rm{t}}}}}}},{{{{{{\rm{h}}}}}}}_{{{{{{\rm{t}}}}}}}\right]$ (*x*, *y* are coordinates of the upper-left corner and *w*, *h* are the width and height of the box, respectively), the ground-truth regression values are determined as ${{Rgt}}_{k}=[\frac{{x}_{t}-{x}_{a}}{{x}_{a}},\frac{{y}_{t}-{y}_{a}}{{y}_{a}},{{{{{\rm{ln}}}}}}\frac{{w}_{t}}{{w}_{a}},{{{{{\rm{ln}}}}}}\frac{{h}_{t}}{{h}_{a}}]$.

The regularization term is a squared L2-norm (or MSE loss with zeros) on ANN extracted features. With *i* iterates through all elements of the 3-D feature vector *F*_*i*_, the regularization term is defined as ${L}_{R}=\frac{{\varSigma }_{i}{F}_{i}^{2}}{{N}_{i}}$.

#### Phase 2: the “where” pathway

The SNN-part is trained with gradients back-propagated from the *L*_*C*_ and *L*_1_ identical to those in phase 1, and an MSE loss *L*_*F*_ between the predicted feature ${F}_{{{{{{\rm{p}}}}}}}\left(t+\Delta t\right)={F}_{a}\left(t\right)+\triangle {F}_{s}\left(\triangle t\right)$ and ground-truth feature ${F}_{a}\left(t+\Delta t\right)$ extracted by the ANN from the frame at corresponding time *t* + Δ*t*:

13 $${L}_{F}\left(t,\triangle t\right)={\sum }_{i}\frac{\triangle {F}_{s}^{i}\left(\triangle t\right)-({F}_{a}^{i}\left(t+\triangle t\right)-{F}_{a}^{i}\left(t\right))}{{N}_{i}}{{\mbox{.}}}$$

The *F*_*loss*_ is designed to ensure that the predicted feature at *t* + Δ*t* resembles presumed the ANN-part extracted features if there is a frame available to the ANN-part at that time, while the *L*_*C*_ and *L*_1_ force it to generate a better tracking result than features from a template frame.

#### Evaluation performance of the HSN based on neuromorphic hardware

To facilitate practical applications, we implemented the HSN based on neuromorphic Tianjic chips^20^. We compared the performance of ANNs, SNNs, and HNNs based on Tianjic chip implementations. SNNs can achieve a higher tracking speed (6613.8 FPS) and lower power consumption (106 μJ/inference) than the ANN; otherwise, the ANN had higher tracking accuracy but suffered from worse power consumption (272 μJ/inference). Conversely, our HSN achieved a balance of efficiency and accuracy. As Table 1 shows, the HSN inherited the advantages of the ANN and SNN, leading to its simultaneous improvement in efficiency (5952.4 FPS speed and 129 μJ/inference power efficiency) and higher performance. Notably, an analysis base on a general-purpose device (GPU or CPU) can be found in Supplementary Material.

**Table 1:** Comparison of computational costs among ANNs, SNNs, and HNNs.

Networks	Operations (10^6^ × OPS)			Power consumption (μJ/inference)	Peak (fps)
	#Add.	#Mul.	#Logic and.		
ANNs	3600	3580	\	272	546.1
SNNs	377	44.5	355	106	6613.8
HNNs	707	372	355	129	5952.4

### Details of the hybrid modulation network

#### The MCL tasks

MCL requires agents to learn multiple similar sub-tasks sequentially. Thus, it is crucial to exploit prior knowledge of previously learned tasks and to avoid catastrophic forgetting. In the HMN, the backbone network and HUs generate high thresholds to inhibit specific neurons in the branch network according to the similarity of the sub-tasks, thus activating similar subnets to perform similar sub-tasks. This mechanism prevents the branch network from catastrophic forgetting and enhances the parameter reuse of the branch network, which means that the HMN is not a trivial bag of subnets.

#### ANN-based backbone network

**Fig. 5:**
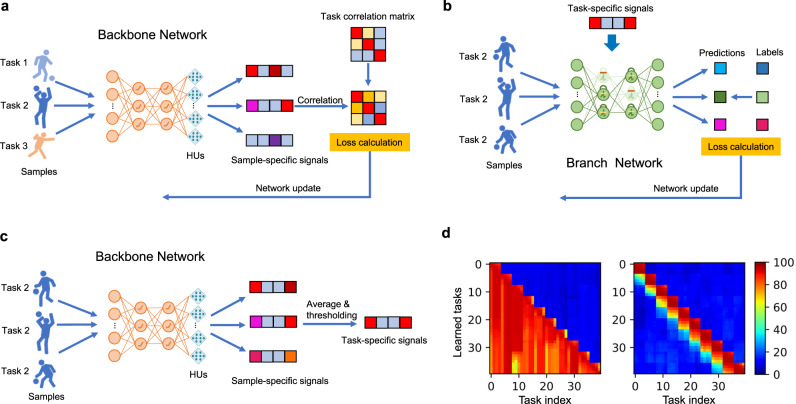
The schematic of HMN. **a** The training framework of ANN-based backbone network. **b** The training framework of an SNN-based branch network for a specific task. **c** The mechanism of generating task-specific signals based on sample-specific signals. **d** The comparison of test accuracy of HMN (left) and SNN (right) on individual tasks vs. the number of learned tasks.

The function of the backbone network is to extract task-related context information. The HUs installed at the backend of the backbone network generate a specific threshold modulation signal for each sub-task according to the task-related information, wherein the modulation signal is a vector and its size is equal to the number of hidden neurons of the branch network. The HUs are jointly trained with the backbone network. The training framework is illustrated in Fig. 5a. The backbone network and HUs, parameterized by *θ*_*a*_, are trained by using the stochastic gradient descent method by minimizing the objective function as follows:

14 $$\mathop{{{{{{\rm{min }}}}}}}\limits_{{\theta }_{a}}{{||}{S}_{{{{{{\rm{ta}}}}}}}-{S}_{{{{{{{\rm{V}}}}}}}_{{{{{{\rm{th}}}}}}}}{||}}_{2}^{2}-\lambda {S}_{{{{{{\rm{ta}}}}}}}\cdot {S}_{{{{{{{\rm{V}}}}}}}_{{{{{{\rm{th}}}}}}}}+\mu \,{{{{{\rm{max }}}}}}\left(\rho -{{||}{S}_{{{{{{{\rm{V}}}}}}}_{{{{{{\rm{th}}}}}}}}{||}}_{1},0\right),$$

where *S*_ta_ and ${S}_{{V}_{{{{{{\rm{th}}}}}}}}$ are the similarity scores between different tasks or modulation signals, respectively. The above objective forces the backbone network and HUs to generate modulation signals according to the similarity of the corresponding tasks. *λ* is the coefficient used to balance these two scores. *ρ* controls the minimum of the sparsity of the modulation signal, and *μ* should be set to a large number. ${S}_{{V}_{{{{{{\rm{th}}}}}}}}$ is calculated as the cosine similarity as follows,

15 $${S}_{{V}_{{{{{{\rm{th}}}}}}}}\left({{{{{{{\rm{V}}}}}}}_{{{{{{\rm{th}}}}}}}}_{i},{{V}_{{{{{{\rm{th}}}}}}}}_{j}\right)=\frac{{{V}_{{{{{{\rm{th}}}}}}}}_{i}^{T}{{V}_{{{{{{\rm{th}}}}}}}}_{j}}{{{||}{{{{{{{\rm{V}}}}}}}_{{{{{{\rm{th}}}}}}}}_{i}{||}}_{2}{{||}{{{{{{{\rm{V}}}}}}}_{{{{{{\rm{th}}}}}}}}_{j}{||}}_{2}}$$

where ${{{{{{{\rm{V}}}}}}}_{{{{{{\rm{th}}}}}}}}_{i}$ and ${{V}_{{{{{{\rm{th}}}}}}}}_{j}$, which range from 0 to 1, are the modulation signals of task *i* and task *j*, respectively. The calculation of *S*_ta_ is an open problem and we provide a feasible definition in this demonstration. We examine the performance of the HMN on the N-MNIST dataset. Different tasks are different spatially permuted versions of the N-MNIST, and thus determined uniquely by the permutation index. We define the similarity between different tasks based on the Hamming distance between the corresponding permutation indices as follows:

16 $${S}_{{{{{{\rm{ta}}}}}}}\left(i,j\right)=1-{{{{{\rm{hamming}}}}}}({p}_{i},{p}_{j})$$

where _*pi*_ is the permutation index of task *i*.

We average the data along the time dimension before feeding it into the backbone network. The backbone network and HUs are trained on all tasks before guiding the branch network to conduct continual learning. After they are trained, the backbone network and HUs generate modulation signals of individual samples of different tasks. We average the modulation signals of all samples in a task and binarize them as the modulation signal of the task. As presented in Fig. 5c, this procedure can be formalized by the following equation:

17 $${{V}_{{{{{{\rm{th}}}}}}}}_{i}={{{{{\rm{max }}}}}}\left({{\mathbb{E}}}_{\left(x,y\right)\in {{{{{{\mathscr{D}}}}}}}_{i}}\left[{{{{{\rm{HU}}}}}}[{{{{{\rm{ANN}}}}}}\left(x\right)]\right]-\frac{1}{2},0\right).$$

#### SNN-based branch networks

The SNN is built by an iterative LIF model and trained by spatiotemporal backpropagation methods as described in Eq. (3) with g(u) = u and ${f}_{s}({{{{{\rm{u}}}}}})={{{{{\rm{H}}}}}}({{{{{\rm{u}}}}}})$. The overall training framework is illustrated in Fig. 5b. For each sub-task *i*, the network parameters *θ*_*s*_ are optimized by

18 $$\mathop{{{{{{\rm{min }}}}}}}\limits_{{\theta }_{s}}{{\mathbb{E}}}_{\left(x,y\right)\epsilon {{{{{{\mathscr{D}}}}}}}_{i}}{{{{{\mathscr{L}}}}}}\left[{{{{{\rm{SNN}}}}}}(x{{{{{\rm{;}}}}}}{\theta }_{s}),y\right]$$

where ${{{{{{\mathscr{D}}}}}}}_{i}$ denotes the datasets of task *i* and ${{{{{\mathscr{L}}}}}}$ denotes the softmax cross-entropy loss. In the HMN, the SNN is trained sequentially on sub-tasks. The thresholds of spiking neurons are modulated by an additive variable generated by the backbone network and HUs. The additional update rule for the threshold when performing sub-task *i* is as follows:

19 $$\widetilde{v}=\left(1-{V}_{{{{{{\rm{t}}}}}}{{{{{{\rm{h}}}}}}}_{i}}\right){{{{{{\rm{v}}}}}}}_{T}$$

where v_*T*_ is a scale factor and typically set to a large number. $\widetilde{v}$ is the modulated threshold that can regularize the dynamics of the network. Using this modulation mechanism, the neuronal excitability of the SNNs can be controlled by the backbone ANNs according to the features of different sub-tasks.

To demonstrate the capability of the HMN in handling similar tasks, we generated sub-tasks in a grouping manner; that is, we set the task similarity in the same group to a high value and the task similarity among different groups to a low value. Specifically, we generated 175 groups of tasks, and there were 4 tasks in each group. We used the tasks in the top 165 groups and half of the tasks in the last 10 groups to train the backbone network and installed HUs. With the modulation, the branch network performed continual learning on the last 10 groups of tasks. Note that the backbone network was not trained on half of the tasks in the last 10 groups. This setting tested the generalization ability of the backbone network, thereby demonstrating the potential of the HMN for practical applications. We adopted an ANN with the structure 34 × 34 × 2 − 512 − 512 and an SNN with the structure of 34 × 34 × 2 − 152 − 152 − 10. We installed 1024 HUs with 512 inputs at the backend of the ANN. More details of the training setup are provided in Supplementary Material.

A comparison of the test accuracy of the HMN and SNN is shown in the Fig. 5d. The accuracy of the SNN on learned tasks decayed rapidly after learning new tasks but that of the HMN remained nearly constant, which indicates that forgetting during continual learning was largely alleviated in the HMN.

### Details of the hybrid reasoning network

#### ANN-based feature extractors

The visual extractor consists of a mask RCNN and a PropNet. The Mask RCNN extracts the color, shape, material, and mask of each object; then the PropNet predicts the motion trajectory of objects according to the output of the Mask RCNN. For predictive and counterfactual questions, the PropNet predicts possible events as well. The language extractor is an SGM^33^, which translates questions into sequential instructions.

#### SNN-based symbolic analyzer

The network is composed of two types of integrate-and-fire neurons used for representing different objects and attributes in the scenario. The first is the representative neuron used for symbolic representations of objective surroundings at different abstract levels, such as red, cube, shape, and collision. The second is the functional neuron used for manipulations of symbols in the reasoning process, such as inhibition, copy, filtering, and ordering. The connecting edges mimic working memory that determines the semantics of the reasoning process, including recalled long-term memory and perceived external information. Long-term memory describes prior knowledge between concepts, such as “red is a kind of color”, and thus forms an abstract semantic structure. The perceived information describes specific relation of concepts for the current environment, such as “the cube is red”, and thus fulfills the semantic structure with arguments. With integrate-and-fire dynamics, the SNN runs symbolic reasoning operations under the instruction of external stimuli. For instance, synchronized stimuli on both “object-inhibition” neurons and “red” neurons will deactivate “object” neurons without the “red” property, indicating filtering object by red color. When receiving the inquiry command, the SGM firstly converts questions into sequential reasoning instructions. Then a designable HU transforms input signals into spike trains, and activates the corresponding neuron nodes in turn. Through the constructed connection relationship, the activating neurons will emit spikes and propagate other adjacent neurons that satisfy the firing conditions, thereby implementing a series of basic logical operations (see Table 2) and performing the entire reasoning process.

**Table 2:** Basic operations supported by HRN.

Objects	Events
**Object-Excitation**	**Event-Excitation**
raise membrane potential (depolarization)	raise membrane potential (depolarization)
**Object-Inhibition**	**Event-Inhibition**
reduce membrane potential (hyperpolarization)	reduce membrane potential (hyperpolarization)
**Object-Copy**	**Event-Copy**
back up the firing state of object neurons	back up the firing state of event neurons
**Object-Self-inhibition**	**Event-Ancestor**
inhibit firing objects after 2-time steps	activate events that influence the firing events
**Object-Attend**	**Event-Attend**
activate objects that attend firing events	activate events that firing objects attend
**Object-Moving**	**Event-Before**
inhibit stationary objects of every firing events	activate earlier events of firing events
**Object-Stationary**	**Event-After**
inhibit moving objects of each firing events	activate later event of firing events
**Object-Shape**	**Event-Start**
filter by or get the shape	the beginning of the video
**Object-Material**	**Event-End**
filter by or get the material	the end of the video
**Object-Color**	**Event-First**
filter by or get the color	deactivate all firing events except the first one
	**Event-Second**
	deactivate all firing events except the second one
	**Event-last**
	deactivate all firing events except the last one

#### Designable and learnable Hus

There are two types of perceived information sent to the SNN: static and dynamic. Static information can be extracted from a single frame of image in the scene, including the color, material, shape, and position of objects. Dynamic information is more complex. It needs to be obtained based on the continuously changing characteristics of objects and can only be extracted from several frames. Moving speed or a collision between objects are two examples of dynamic information. Considering the different characteristics of these two types of information, HRN introduces designable HUs and learnable HUs to extract symbolic representations of them, respectively. In particular, the designable HU directly converts static information into spike signals by stimulating corresponding spiking neurons, and then constructs the connection relationship between objects with Hebbian learning rules. In the CLEVRER task, the designable HU first associates the nodes of observed objects in the current frame with the nodes of the attribute concept and then establishes connections based on their firing state. For example, if it is detected that object “A” in the scene is red, node “red” and node “object A” are connected by a positive weight. To accurately capture the dynamic information in the scene, the HRN further introduces learnable HUs with independent learning stages to extract symbolic features from multiple adjacent frames. The learnable HU is constructed to determine the condition under which a collision occurred. Because the event representing “object collision” is labeled in the training set of CLEVRER, we use supervised learning on a one-dimension UNet with two MLP heads. The inputs are moving trajectories of two objects (linearly interpolated for missing frames). The UNet abstracts multi-scale temporal features and mixes them into an intermediate representation of the same length. Then the two MLP heads predict if the two objects collide and when the collision happens. We use cross-entropy (CE) and mean square error (MSE) to calculate the loss of two heads, respectively, and use Adam to minimize the combination loss (5*CE_loss + MSE_loss).

#### Experimental configurations on the CLEVRER dataset

We applied our model to CLEVRER datasets and validated its performance over four types of tasks: descriptive, explanatory, predictive, and counterfactual tasks. In the robustness experiment, we adjusted the threshold for collision detection to generate abnormal data for robustness validation. Specifically, in collision detection, the HRN sets the collision detection to a given threshold to determine whether neighboring objects will collide in the next moment. When the object is sufficiently fast and the two objects are sufficiently close, the HRN detects that a collision would occur. Based on this, we controlled the probability of generating abnormal data by adjusting the detection object judgment threshold. It allowed the HU to misjudge non-collision frames as collision frames, thereby generating abnormal information for robustness testing. We controlled the different detection thresholds and show the corresponding results in Fig. 4d.

## Supplementary information

Supplementary Information

## Data Availability

Implementation of HNNs is made public together with the publication of this paper https://github.com/IbrahimYang/Hybrid-neural-networks.

## Code availability

All data used in this paper are publicly available and can be accessed at the following link after the paper is published. https://github.com/IbrahimYang/Hybrid-neural-networks.
