# Supplementary material for: A framework for the general design and computation of hybrid neural networks
Source: Nat Commun. 2022 Jun 14;13:3427. doi: 10.1038/s41467-022-30964-7 (PMC9198039; doi:10.1038/s41467-022-30964-7)
Supplement: Supplementary file 1 — Supplementary Information [file 41467_2022_30964_MOESM1_ESM.pdf]

A Framework for the General Design and Computation of Hybrid Neural Networks

Supplementary Note 1: Experimental Settings and Dataset of the Hybrid Sensing Network

Benchmark datasets for hybrid streaming perception. To demonstrate the superiority of HSN in hybrid tracking, we developed a data generation method that can generate a hybrid dataset simultaneously containing the stationary-based (parvocellular pathway) and transient-based (magnocellular pathway) signals[1,2], shown in Supplementary Figure. 1a. To this end, we generated DAVIS[3] streams using intensity frames from traditional video-based datasets, NfS[4], VOT[5], and CLEVRER[5]. As shown in Supplementary Figure. 1b, we use the two-phase generation method, including a data interpolation phase and spikes generation phase.

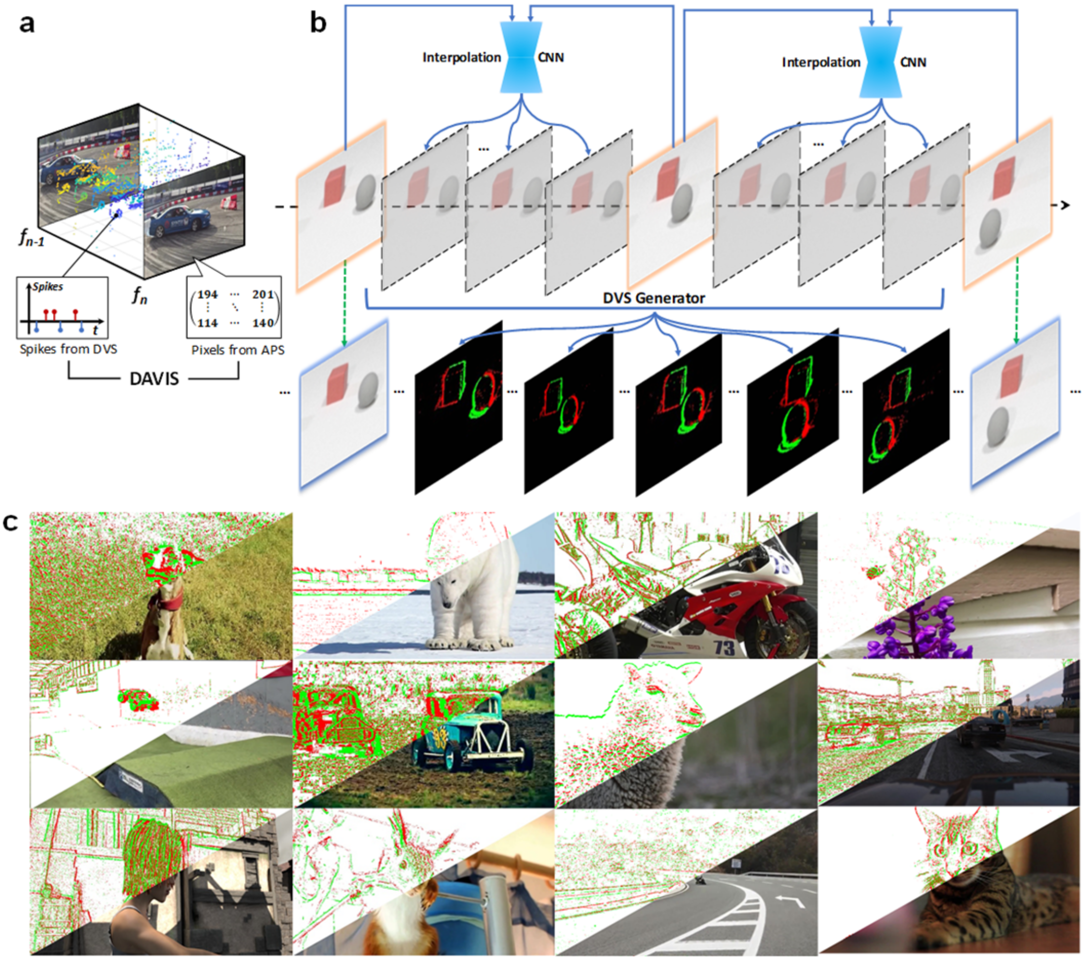

Supplementary Figure 1 | Overview of the dual retinal pathway dataset. **a**, The dual visual pathway and basic model of hybrid visual streaming. **b**, The generation framework for the DAVIS simulator. **c**, Side-by-side comparison of simulated data with frame-based data.

Data interpolation phase. We input adjacent pairs of images (I_{n-1}, I_{n+1}) to the CNN and predict the bi-directional optical flow (op_{n-1}, op_{n+1}) to the last and next frames. Then flow vectors are linearly separated with an arbitrary time. Finally, we warp the flow information to predict the inter-frame I_n. In the training process, we randomly pick up the adjacent three frames from video sequences and extract the middle frame as the ground truth. Therefore, we choose the L_1 -norm-based pixel-to-pixel loss $L_1 = ||I_n - I_{gt}||_1$ and perception loss with a pre-trained VGG[6]network as our loss function.

Spike generation phase. The DAVIS data jointly include asynchronous spikes from DVS and synchronous frames from APS. In the APS part, we directly extract non-interpolation frames in t_f with a fixed temporal interval Δt_f and contained m frames. In the DVS part, we denote one DVS event by a quaternion $\{x, y, p, t_e\}$, where x and y denote the pixel coordinates of the event, $p \in \{-1, +1\}$ denotes the polarity of the event, t_e denotes the timestamp of the event. The DVS spike will fire when the brightness changes higher than a pre-set threshold θ within the minimal temporal resolution Δt_e. We define brightness changes as $\Delta I(x, y) = I_n(x, y) - I_{n-1}(x, y)$, from the interpolation stage. After that, we regard the $\Delta I(x, y)$ as the firing rate $f_e(x, y)$ of the (x, y) pixel, as same as the number of events between I_n and I_{n-1}. Since DVS has random temporal jitter between I_n and I_{n-1}, we use the Poisson process to generate spikes with random timestamps.

Performance definition. We adopted the widely accepted "streaming accuracy"[7], which fully considers the real latency in the hardware processing pipeline and the limitation of computational resources. The traditional benchmark ignores latency effects due to hardware processing and only compares the performance with an offline approach, where $error = \|\hat{y}(t) - y(t)\|_2$. Here, the sensor inputs, network outputs, and ground-truth at time-stamp t are denoted by $x(t), \hat{y}(t), y(t)$, respectively. It can be noticed that because the hardware processing consumes time T_C , for example $T_{ANNs}\ or\ T_{SNNs}$, the environment has changed from $y(t)$ to $y(t + T_C)$ when the processing completes. Therefore, to fairly judge the ability of an autonomous agent (re)acting in the real environment, we need to incorporate the $error$ equation from $\|\hat{y}(t) - y(t)\|_2$ to $\|\hat{y}(t) - y(t + T_C)\|_2$.

Parameter settings. CLEVRER dataset contains 10000 training videos and 5000 test videos. Each frame is resized from 320*480 to 128*192 and padded to 232*296 by symmetrically padding zeros before feeding into the network. As shown in Supplementary Table 1, we adopt two parallel ANNs and SNNs with the same ResNet-

22 backbone to extract features from APS and DVS, respectively. The features extracted from DVS frames are considered deviations from APS features and hence added to the template APS frame feature, resulting in feature predictions at a time between two consecutive APS frames. Then, target features and predicted features from ANNs and SNNs are merged in HUs to calculate bounding box prediction results. The HUs use 4 conv2d layers to extract classification and regression results of each anchor, 6 anchors are used for each location. Similar ideas can be found in RPN[8] (region proposal network), but the main difference is that HUs in HSN realize the fusion of different time-scale and precision information. During training, a batch size of 40 was used for the ANN part and a batch size of 5 was used for the SNN part. Both ANN, SNN, and HUs parts use an Adam optimizer with a learning rate of 0.0001 for gradient descent.

Supplementary Table 1 Network structure of HSN

Layer name	Structure of the ANN-part	Structure of the SNN-part
Conv1	$7 \times 7, 64, stride\ 2$	$7 \times 7, 64, stride\ 2$
Conv2	$\begin{bmatrix} 1 \times 1, 64 \\ 3 \times 3, 64 \\ 1 \times 1, 256 \end{bmatrix} \times 3, stride\ 2$	$\begin{bmatrix} 1 \times 1, 64 \\ 3 \times 3, 64 \\ 1 \times 1, 256 \end{bmatrix} \times 3, stride\ 2$
Conv3	$\begin{bmatrix} 1 \times 1, 128 \\ 3 \times 3, 128 \\ 1 \times 1, 512 \end{bmatrix} \times 4, stride\ 2$	$\begin{bmatrix} 1 \times 1, 128 \\ 3 \times 3, 128 \\ 1 \times 1, 512 \end{bmatrix} \times 4, stride\ 2$
HUs	$\begin{bmatrix} 3 \times 3, 512 \\ 3 \times 3, 512 \\ 3 \times 3, 512 \times 6 \times 2 \\ 3 \times 3, 512 \times 6 \times 4 \end{bmatrix}$	

Supplementary Note 2: Experimental Details of the Hybrid Modulation Network

Parameter settings. N-MNIST dataset contains 60000 training samples and 10000 test samples. The spatial size is 34×34 and the time depth is 10. We adopt an ANN with the structure of $34 \times 34 \times 2 - 512 - 512$ and an SNN with the structure of $34 \times 34 \times 2 - 512 - 512 - 10$. 1024 HUs with 512 inputs are installed at the backend of the ANN. The ANN is trained for 30 epochs by SGD without momentum. The initial learning rate is 0.02 and attenuates to 0.002 after 25 epochs. ρ is set to 10 and μ is set to 0.05. The SNN is trained for 10 epochs for each task by SGD. The initial learning rate is 0.05 and attenuates to 0.005 after 5 epochs. The threshold of the SNN is fixed as 0.2. The batch size is set to 200 for all experiments.

Scaling analysis. To demonstrate the scaling advantage of HNNs over both SNNs and ANNs, we have conducted an empirical study about the HMN on continual learning tasks. Considering the spike-based neuromorphic applications have achieved a wide range of success, we adopt DVS data as the input data, and build an SNN network with typical advanced continual learning techniques (Xdg) for comparisons. On this basis, we conduct a series of experiments of learning various numbers of tasks with different network capacities measured by the number of hidden neurons. The performance measured by the mean accuracy of all tasks after sequential learning is reported in the Supplementary Figure. 2.

Here we would like to summarize three important observations as follows:

1. The proposed HMN's performance exceeds those of the SNN models in all controlled experiments with different settings.
2. When the task number is small, such as 32, the smaller the network capacity, the larger the performance gap.
3. When the task number is large, such as 128, the larger the network capacity, the larger the performance gap. Even with enough network capacity, the performance of the single SNN saturates and is lower than that of HNN by a considerable margin.

To investigate the effectiveness of HMN quantitatively, we analyzed the reuse ratio of parameters of different models in the setting of 128 tasks with 16384 channels. The reuse ratio is defined by the ratio of parameters shared between different tasks. A large reuse ratio for uncorrelated tasks indicates the parameter interference, resulting in catastrophic forgetting, while a small reuse ratio for highly correlated tasks results in inefficiency of model capacity utility. Results demonstrate that, for both highly correlated tasks and uncorrelated tasks, the average reuse ratio is about 0.10 in the SNN model. In contrast, in the HNN model, the average reuse ratio is about 0.50 for highly correlated tasks, and 0.03 for uncorrelated tasks. These results validate that HMN can effectively alleviate the interference between the parameters allocated to perform uncorrelated tasks, and reuse the parameters for correlated tasks, thus leading to superior performance. The scaling advantage of the HMN on continual learning tasks is facilitated by the additional model complexity of HNN, which is brought by the hierarchical multi-network architecture and the diverse parameter modulation between heterogeneous networks.

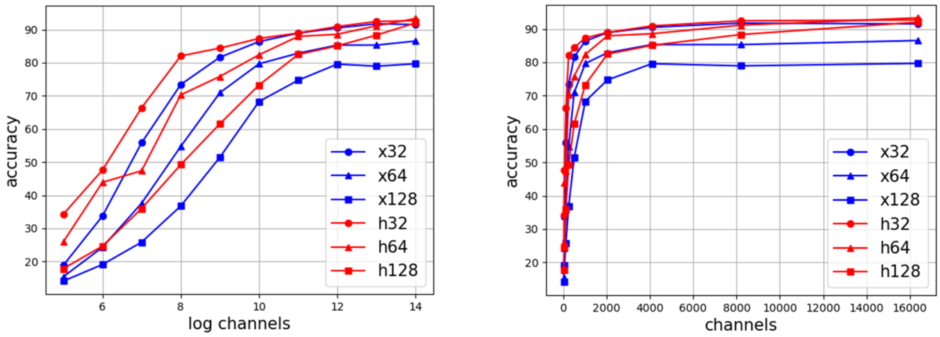

Supplementary Figure 2 | Mean accuracy of different models on all learned tasks vs. the numbers of the hidden neurons. For better visualization, these figures are presented in different x-coordinate scales (left: logarithmic, right: linear). X32 refers to the SNN model on 32tasks, and h32 refers to the HNN model on 32tasks.

Comparison with ANN-only and SNN-only implementations. The hybrid modulation network utilizes the powerful data fitting ability of ANN and the rich spatiotemporal dynamics of SNNs, enabling multi-network to perform hierarchical processing tasks of meta-continuous learning. This experimental setup aims at demonstrating the importance of hybrid modulation in the coordination of hybrid multi-networks. Although it can still work if the ANN modulator is substituted by a rate-coding SNN modulator with a similar architecture, the efficiency and performance will significantly degrade. To verify the above claim and demonstrate the effectiveness of HNN, we have conducted extensive contrast experiments. The experimental settings, results, and analysis are presented in Supplementary Table 2.

Supplementary Table 2 comparison of ANN-only and SNN-only implementations in HMN

Model	Number of parameters	Input channels (backbone network	Input channels (branch network	Mean accuracy
ANN-only	1.97M+1.45M	Take the mean along the time dimension	Take the mean along the time dimension	76.86±1.37
SNN-only	1.97M+1.45M	Input iteratively in 10 time steps	Input iteratively in 10 time steps	60.48±5.34
HNN	1.97M+1.45M	Take the mean along the time dimension	Input iteratively in 10 time steps	80.21±2.36

For the ANN-only implementation, we replace the branch network of the HMN with an ANN network with the same architecture. According to the usual practice, we take the mean of the inputs along the time dimension before sending the samples to the branch network. For the SNN-only implementation, we replace the backbone network of HMN with an SNN network with the same architecture. The inputs are fed into the network in 10 time steps. As shown in the Supplementary Table 2, the mean accuracy of HMN on continuous learning 40 tasks exceeds that of ANN-only and SNN-only implementations by a large margin.

For the ANN-only implementation, it is difficult to discriminate the fine-grained timing feature of the inputs for the branch network implemented by an ANN due to the averaged inputs along the time dimension. For the SNN-only implementation, it is difficult to fit the task-level context information for the backbone network implemented by an SNN due to the immature optimization algorithm of SNNs. Therefore, these implementations result in performance degradation. The experimental results verify that the HNN can utilize the advantages of both ANNs and SNNs to perform appropriate tasks.

Supplementary Note 3: the Universal Approximation of Hybrid Units

The approximation capability of ANNs has been studied based on the universal approximation theorem developed in the 1990s[9, 10, 11]. Hornik et al.[9] proved that there exists a three-layered feedforward network with bounded and non-constant semi-linear functions, e.g. sigmoidal function, which can approximate any continuous function arbitrarily well. Blum et al. [10] and Kolmogorov et al. [11] also provide a constructive proof on the approximation property.

At the same time, it has frequently been conjectured that generic computations by neural circuits are not digital, and are not carried out on a static input but rather on functions of time[12]. Thus, if we accept the assumption that the firing times of neurons in a biological neural system encode relevant information, there are still many possible spiking coding schemes that encode information in a way different from that of rated-based traditional networks, such as first-to-spike coding, temporal interval coding, etc. Along this line, previous work has studied the approximation capabilities of SNNs with some specific coding paradigm or some specific computational framework[13, 14]. However, it still lacks a theoretical analysis of the universal approximation capability of such versatile spiking coding.

Furthermore, hybrid information conversion is a map from a set of spiking functions $s(t)$ to a set of real numbers. In principle, SNNs can implicitly encode the information in the spike function $s(t)$ in an arbitrary manner. However, to the best of our knowledge, it is unknown whether for any spiking coding, it has a general model to approximate any arbitrary filter with a given precision.

The problem can be divided into two cases: (1). If we know the specific coding method φ (i.e., filtering methods), we can adopt designable methods to construct HUs. (2). If the encoding method φ for the information I is unknown, can the HNN framework capture the effective information contained in it?

We would like to point out that it can use learnable HUs for general approximation. Next, we argue that some modified learnable HUs can approximate arbitrary given filters to any desired degree of precision. Our proof yields two following assumptions,

(1) Positive minimum spiking interval. It exits a minimum interval between any two spikes in one spike train $S_i(\{t_k^f\})$.

$$\Delta t^* = inf\left\{|t_i - t_j|\middle|\forall t_i, t_j \in \{t_k^f\}\right\} > 0 \tag{1}$$

(2) A compact temporal domain (i.e., $\exists T \in R, \forall t_i \in \{t_k^f\}, t_i < T$) . That is to say, we mainly consider the case that spike firing time happened in a compact temporal domain.

The main goal is to illustrate that for any filter (i.e., spiking decoding function), some suitably modified HUs can approximate the filter with any desired degree of precision. To this end, we first formalize the universal approximation theory of HUs as below,

Theory 1. *Given an error gap* $\epsilon \geq 0$, *for any function* $\varphi: S^m(t) \to R^n$, *there exists a composite function* $\varphi_\epsilon: S^m \to R^n$ *with representation*

$$f_\epsilon = F * H \tag{2}$$

*where F denotes a composable affine map consisting by a three-layer feedforward network with bounded and non-constant semi-linear functions used for domain transformation, H denotes a composable affine map used for the information conversion between time domain and amplitude domain, and * denotes component-wise composition, such that the approximation bound*

$$||f_\epsilon - f|| < \epsilon \tag{3}$$

holds for any arbitrarily small ϵ.

To accomplish this proof, we first point out that the information capacity of spike trains can be approximated by a finite set of spline basis functions and a finite-dimensional representation matrix. Then we use the existing approximation theory of ANNs or SNNs to support the theoretical effectiveness of HUs.

At the first step, we prove that for any given filter (i.e., spiking decoding function) φ yielding a positive minimum spiking interval, there exists a piecewise linear function $\varphi_{\epsilon 1}$with a finite value that can approximate the φ under the approximation precision ϵ_1 as below.

Lemma 1 (The universal approximation capability of HUs to any decoding filtering). *Given an error gap* $\epsilon_1 = \frac{\epsilon}{2} > 0$, *for any filtering function* $\varphi(s(t))$ *where spike trains* $s^m(t)|: R \rightarrow R$ *defined in a compact temporal space, there exits some suitable modified function* $\varphi_{\epsilon 1} := H * Q|S^m \rightarrow R^n$

$$\|\varphi_{\epsilon 1} - \varphi\| < \frac{\epsilon}{2} \tag{4}$$

Proof.

We begin by setting the spiking function as the unit step function (i.e., piecewise constants described by the Type A spiking neuron defined in Wolfgang et al. [14])

$$k(t - t_i) := \frac{1}{\alpha}\mathbf{1}_{[c_i, c_i+\alpha)}(t) \tag{5}$$

where $\mathbf{1}_{[\boldsymbol{a},\boldsymbol{b}]}(\mathrm{t})$ denotes the indicator function yielding

$$\mathbf{1}_{[\boldsymbol{a},\boldsymbol{b}]}(\mathrm{t}) = \begin{cases} 1, t \in [a, b] \\ 0, t \notin [a, b] \end{cases} \tag{6}$$

In particular, when $\alpha \rightarrow 0$, the A-type spiking functions can approach commonly-used delta functions $\delta(t)$.

We denote the minimum firing interval by dt and set the first spike firing time as the reference time (i.e., $min\{t_i^f \,|\, i = 1,2, \dots, f = 1,2, ..\} = t_0$). Let the interval [0, T] be covered by N ordered subintervals with pairwise disjoint interiors

$$\begin{gathered} [c_i, c_{i+1}] := [i * T/N, (i + 1) * T/N) \\ t_0 = c_0 < c_1 < \cdots < c_N = T \end{gathered} \tag{7}$$

On each of these i pieces of $[c_i, c_{i+1}]$, we want to define the pairwise basis function

$$\omega_i = \frac{1}{\alpha}\mathbf{1}_{[c_i, c_{i+1})} \tag{8}$$

Then for any spike train $s_\mathrm{i} = \sum_i \kappa(t - t_i^f)$ with spiking firing function κ, it can be approximated by $\tilde{s}_\mathrm{i}$

$$\tilde{s}_\mathrm{i} := \begin{bmatrix} 1 & \dots & 1 \\ 0 & 1 & 0 \\ 0 & \dots & 0 \end{bmatrix}_{m \times N} [\omega_1, \omega_2, \omega_3, \dots., \omega_N] := B * C^T \tag{9}$$

Analytically, the approximate error is caused by the difference between the arbitrary spike firing time t_i^f and the most relevant basis function time $\frac{\mathrm{c_i + c_{i+1}}}{2}$. Note that when the spike meets the assumption of the minimum firing interval, each neuron fires at most $n_0 = \lceil T/\Delta t^* \rceil$ times in a given time domain. On this basis, it can be seen from the construction of basis function that as $N = \frac{\epsilon}{\alpha m n_0} + 1$ is large enough, the approximation error satisfies

$$\int_{t_0}^{t_0+T} |s - \tilde{s}_\mathrm{i}| dt = \sum_{t_i} \int_{t_i}^{t_i+\Delta t^*} |s_i - \tilde{s}_\mathrm{i}| dt < n_0 \frac{\epsilon}{2m} \tag{10}$$

For s^m, it can be approximated by a binary matrix $B \in R^{m \times k}, \forall b_{ij} \in \{0,1\}$ multiplied with a set of basic functions $C^T = [\omega_1, \omega_2, \omega_3, \ldots., \omega_N]$ which yields

$$Rank(B) \leq \min\{m, n_0\} \quad (11)$$

The above results indicate that for given the spike trains with any given precision, it can be approximated by a finite Euclid space. It exits a finite set of basis function representation methods to approximate any given spiking representation by $\varphi_{\epsilon 1}$ with arbitrary precision. Such approximation for any spiking decoding function can be implemented and identified as H in the HUs. Then we can follow the Weierstrass approximation theorem to find an approximation $\varphi_{\epsilon 1}$ to approximate the decoding function φ_ϵ.

Given the above results, for any spiking function, in principle, it can be represented by a finite real space R^k to approximate the signal representation with a given precision. By using existing theories of universal approximation for NNs, it can be further proved that there exists a composable affine map F to project the information distribution from R^k into R^m under the approximation error $\epsilon/2$

Finally, with the help of triangle inequalities, we can easily get

$$\|f_\epsilon(s) - f(s)\| = \overbrace{\|\varphi_{\epsilon 1} - f_\epsilon(s)\|}^{H} + \overbrace{\|\varphi_{\epsilon 1} - f\|}^{F} \leq \frac{\epsilon}{2} + \frac{\epsilon}{2} = \epsilon \quad (12)$$

The first term $\|\varphi_{\epsilon 1} - f_\epsilon(s)\|$ can be evaluated by above Lemma 1. We can use the existing conclusion of approximate capability in ANNs or SNNs [9-12] to illustrate that

$$\|\varphi_{\epsilon 1} - f\| \leq \epsilon/2 \quad (13)$$

Supplementary Note 4: Details of Bio-plausibility of Hybrid Neural Network Models

Bio-plausibility of the HSN. The P pathway and M pathway are two well-known pathways for visual information processing[15], which have features similar to those of ANNs and SNNs, respectively. Specifically, the P-pathway has a slow conduction velocity and responds strongly to changes in colour, but only responds weakly to changes in contrast[16]. Considering the constraint in the visual system, the transmission of such high-precision information needed for object recognition suffers from low speed. This feature resembles the functional role of ANNs in the HSN. In contrast, the M-pathway has a fast conduction velocity[17] and responds to low-contrast stimuli, but is less sensitive to changes in colour [18]. In this case, the high-speed conduction necessary for sensitive detection sacrifices high precision in terms of colour. This feature matches the role of SNNs in the HSN. To summarize, because of the constraint of accessible computing resources, high precision is needed in detailed object recognition, but recognition may be slower, whereas high speed is essential to detect flying events, but

spatial resolution and colour information must be sacrificed. Dividing raw visual information into two pathways with a distinctive response property and conquering them separately seems to be an efficient strategy to balance the intricate, and even contradictory, requirements of real-world scenarios.

Bio-plausibility of the HMN. The adaptivity of the brain function when it encounters diverse environmental scenarios fundamentally requires neuromodulation, graded and relatively slow changes to fast synaptic transmission and ion channel properties through diffusible signalling molecules [19, 20, 21]. Two parts of neuromodulation, that is slow diffusive modulation and fast synaptic transmission, are comparable to the ANNs and SNNs in the HMN respectively. We explain this point as follows: Consider neuropeptides as an example, a recently discovered fine-grained neuromodulator with higher diversity, selectivity, and specificity[22, 23] . Secreted neuropeptides are thought to persist as long as minutes and diffuse to as broad as hundreds of micrometres[20]. With the recent advent of single-cell and neurotaxonomic methods, a specific prediction has been made that dense intra-cortical neuropeptide modulatory networks may play prominent roles in cortical homeostasis and plasticity[22, 23]. Interestingly, the ANN part in the HMN emerges as a similar fine-grained modulatory dense network with longer persistent activities (operation is slower), broader influence (each ANN unit influences a group of spiking neurons), and higher neuron specificity (modulatory activity is task specific), whose larger spatiotemporal scale activity can adjust the fast transmission property of SNN parts.

Bio-plausibility of the HRN. The reasoning and deducting capability of neural networks implicitly requires symbol-like processing [24]. In increasing numbers of studies, it is suggested that neural systems exploit sparse timing correlated neuronal groups as basic representative units of symbols which can be dynamically associated with form pointers through the Hebbian learning rule[25]. Such a symbol-like representation of variables, roles, or syntax, cannot work alone. It needs to be grounded by values, fillers, or words to fulfil the cognitive, mnemonic and linguistic capabilities of humans[24]. Despite the simplification, the SRN demonstration in this study illustrates how symbol-like processing and its grounding can be realized by SNNs and ANNs, respectively. ANNs play their roles in extracting low-dimensional invariant features with a distributed representation, and SNNs dynamically associate its sparse groups of neurons accordingly. The specific structure of the SRN that is essential for its reasoning capability provides a new hypothesis for the working memory mechanism. For example, it suggests the requirement of a proper transforming mechanism between different sensory cortical areas and prefrontal working memory areas in the brain. Additionally, this demonstration provides a reminder of arguments on the preconfigured versus the

blank slate model of the brain, where backbone connectivity and its emergent dynamics are genetically defined as an existing knowledge base[26].

Supplementary Note 5: Benchmarking Hybrid Units on Classification Tasks

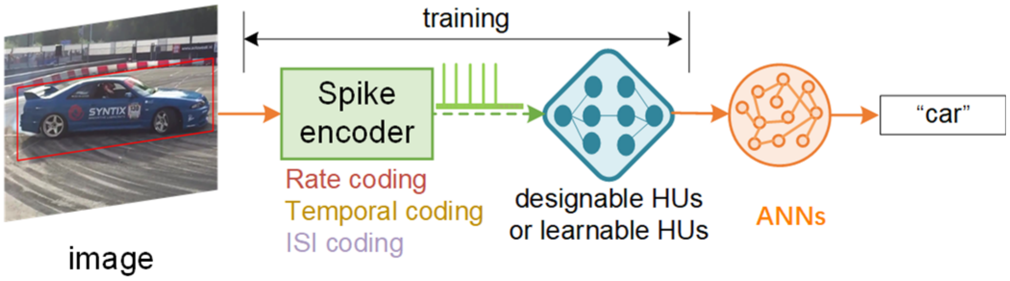

Supplementary Figure 3 | Illustration of the pipeline of the HUs for spike train encoding and decoding. HUs transmit information from ANNs to SNNs. The spike trains are synthesized by classically designed coding schemes such as rate coding.

HUs transmit information from SNNs to ANNs. To demonstrate the adaptivity of learnable HUs, we conducted experiments comparing learnable HUs with classical manual decoding schemes on various unknown spike trains. The pipelines of the experiment are shown in Supplementary Figure 3. The accuracies of networks on classification tasks with different HUs are reported in Supplementary Table 3. The results show that the HNN with learnable HUs can achieve high accuracy under different encoding schemes, indicating that learnable HUs can adaptively decode unknown spike trains. In contrast, the HNN with designed HUs can only achieve the desired performance when the decoding scheme corresponds to the encoder and decoder, but not in other cases.

Supplementary Table 3 Classification accuracies under different settings. We repeat three trials for each model and report the average results (i.e., mean±std).

decoder / encoder	learnable HUs	designable HUs		
		rate decoder	temporal decoder	ISI decoder
learnable encoder	**85.8%±0.2%**	15.1%±0.1%	12.0%±0.1%	16.0%±0.1%
rate encoder	84.5%±0.1%	**85.4%±0.2%**	54.9%±0.5%	71.3%±0.2%
temporal encoder	**85.4%±0.1%**	10.0%±0.0%	85.2%±0.2%	10.0%±0.0%
ISI encoder	80.2%±0.2%	69.3%±0.8%	10.0%±0.0%	**85.2%±0.2%**

Experimental data pipeline: Firstly, we synthesized four spike encoders with two different types to generate spike trains based on the CIFAR-10 dataset. The first type is a classical encoder with rate coding, temporal first-to-spike coding, and inter-spike-interval (ISI) coding, respectively. The second type is a learnable encoder, which

directly learns the coding scheme from the data. Then, we use learnable HUs and the above three designed HUs to decode the generated spike trains. Finally, the decoded results are fed into an ANN-based CNN for classification.

Spike encoder: Suppose the output spiking is denoted by $S(t)$. For the rate encoder, it can be formalized by $\int_0^W S(t)\,dt/W = x$, where x is the value of each pixel, and W denotes the time window function. In our demonstration, all encoders encode information with $W = 100$. For the temporal encoder, the timestep of the first spike is equal to the value of each pixel. For the ISI encoder, the interval of the first two spikes is equal to the value of each pixel. For the learnable encoder, it is a one-layer HUs that can be formalized by $F \cdot H \cdot W(x)$, where W is a rectangle window, H is a trainable tensor with the same dimension of W in the time domain, and the F is a step activation function.

Learnable and designable HUs: The learnable HUs can be formalized by $F \cdot H \cdot W(X)$. In our demonstration, X denotes the encoding spike with 100 timesteps. Therefore, we choose HUs with the dimension of $W = 100$, the dimension of $H = 10$, and the F is a ReLU function. The weights of W and H are trained independently by mutual information with an unsupervised training paradigm. For the image classification task of CIFAR-10, a set of encoding and decoding methods are chosen for each trial of the experiment to transmit the value of each pixel of the image separately. After transmission, a conventional training process of image classification is carried out on the CIFAR-10 dataset. The classification method we used here is an AlexNet-style network, with a 12-layer feature extractor and a 3-layer MLP classifier. During training, trials were repeated three times. The average results and standard deviations are reported in Supplementary Table 3.

Supplementary Note 6: Computational Cost of Artificial Neural Networks, Spiking Neural Networks, and Hybrid Neural Networks on Graphics Processing Units

In this section, we firstly analyze the computational cost of ANNs and SNNs with the same network structure; Then, ANNs, SNNs, and HNNs are deployed on the same hardware to evaluate the execution time. The following discussion focuses on the MLP structure with n-digit integer weights and activations for simplicity. The comparison can be easily extended to other structures, such as convolution or floating-point cases.

For the l_{th} layer of ANNs, the dimension of weight is $N_{l-1} \cdot N_l$, where N_l indicates the neuron number of l_{th} layer. On this basis, we can obtain the computational cost (time cost) boundary $O(N_{l-1} \cdot N_l)$ of an MLP structure. The memory cost (space cost) boundary is $O(N_{l-1} \cdot N_l + N_l)$. If the weights and activations are n-digit numbers, the computation cost of "addition" is $O(n)$, "logic and" is $O(1)$, and "multiplication" is

$O(n^2)$[27]. Therefore, the computational cost C_l^{ANN} and memory access cost M_l^{ANN} can be described by

$$\begin{gathered} C_l^{ANN} \sim O(n^2 N_{l-1} N_l + n N_{l-1} N_l) \\ M_l^{ANN} \sim O(n N_{l-1} N_l + n N_l) \end{gathered} . \tag{14}$$

Due to the binary spike activation, SNNs can bypass most of "multiplication" operations and only have "addition" and "logic and" operations. In addition, the binary spikes are transmitted through an event-driven approach. Benefitting from these features, SNNs can significantly alleviate the bandwidth requirement and reduce the computational cost. Supposing that the firing rate of the l_{th} layer is $R_l \in [0,1]$ in SNNs, the computational cost of "addition" and "logic and" operations follows $O(R_{l-1} \cdot N_{l-1} \cdot N_l)$ for dendritic inputs, and the computational cost of "multiplication" operations follows $O(N_l)$ for membrane potential updates. The memory cost (spatial cost) boundary follows $O(R_{l-1} \cdot N_{l-1} \cdot N_l + R_l \cdot N_l)$. If the weights of SNNs are n-digit numbers, the computational cost C_l^{SNN} and memory access cost M_l^{SNN} can be described by

$$\begin{gathered} C_l^{SNN} \sim O(R_{l-1} N_{l-1} N_l + n R_{l-1} N_{l-1} N_l + n^2 N_l) \\ M_l^{SNN} \sim O(n R_{l-1} N_{l-1} N_l + R_l N_l) \end{gathered} . \tag{15}$$

Here, we assume that both ANNs and SNNs work on the general computing core[28] with a peak computation capacity of α_{max}(operations/s) and a memory bandwidth of β(bit/s). The runtime computing capacity α follows

$$\alpha = min(\alpha_{max}, \beta \cdot operational\ intensity). \tag{16}$$

where the $operational\ intensity$ means operations per byte of data traffic.

Therefore, we can get the time cost of the l_{th} layer ANNs and SNNs follows

$$\begin{gathered} T_l^{ANN} = \frac{C_l^{ANN}}{\alpha} + \frac{M_l^{ANN}}{\beta} \\ T_l^{SNN} = \frac{C_l^{SNN}}{\alpha} + \frac{M_l^{SNN}}{\beta} \end{gathered} . \tag{17}$$

Usually, inputs of ANNs from sensors are uniform with the same time interval T_0 (generally in the APS sensor $T_0 = 30ms$). Due to the synchronous feature of ANNs, it is hard to input data through an event-driven approach (e.g., part of the pixels are input first, and then the remaining pixels are input after several time steps) and the computation of the next layer heavily relies on the results from the previous layer. Therefore, the total time consumption is equal to the maximum time consumption of sensing and computation

$$T_{ANNs} = max(T_0, \sum_{l=1}^{L} T_l^{ANN}) = max(T_0, \sum_{l=1}^{L}(\frac{C_l^{ANN}}{\alpha} + \frac{M_l^{ANN}}{\beta})). \tag{18}$$

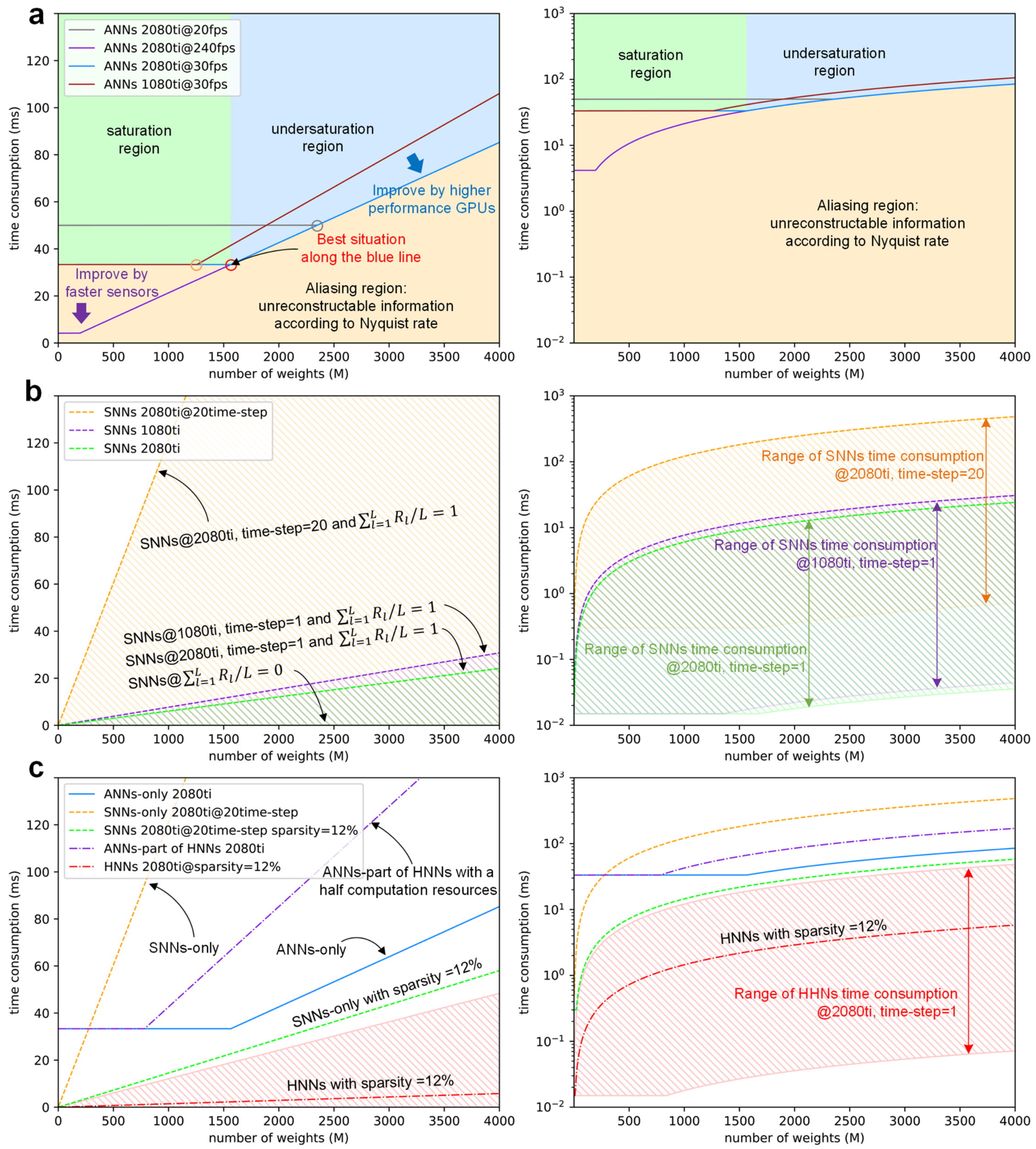

Supplementary Figure 4 | Curves of the time consumption versus the number of weights. a: ANNs, b: SNNs, c: HNNs. The linear-linear plot on the left is to demonstrate the absolute values, while the log-linear plot on the right shows the $\sum_{l=1}^{L} R_l\,/L = 0$. a. Each curve of ANNs contains three different regions: the saturation region (green region), the undersaturation region (blue region), and the aliasing region (yellow region under the curve). The circle refers to the turning point of each curve. b. The dashed line indicates the SNN without any sparsity ($\sum_{l=1}^{L} R_l\,/L = 1$) and the bottom line indicates none of neurons fired ($\sum_{l=1}^{L} R_l\,/L = 0$). Depending on different inputs, the time consumption of SNNs is between the top and bottom lines. c. The solid line represents ANNs, the dashed line represents SNNs, and the dotted line represents HNNs. Notably, GPUs' computation capacity is usually defined by FLOPS (floating-point operations per second), while IPS (instructions per second) are used for CPUs. Typically, the *n-digit* floating-point OPS is equal to *1/n* binary OPS[29] in most GPUs. Also, GPUs usually use Fused multiply-add (one operation in GPUs) instead of calculating "multiplication" first and then "addition"

(two different instructions in CPUs). Therefore, all curves above follow GPUs condition, which is slightly different from the general case.

For ANNs, according to (18), we calculate the time consumption and the number of weights diagram as plotted in Supplementary Figure.4a. The saturation region means that the consumption of the total computation time of ANNs is lower than the sensor, indicating that the hardware doesn't have enough computation resources to process the data as fast as the sensor sampling. The undersaturation region is the opposite case. According to the Nyquist rate, twice the region under the curve is the aliasing region. In tracking and detection tasks, it means even every single recognized position is right, but the trajectory is totally wrong. For most of ANNs, once the model size, computation chip, and sensor are determined, the speed (time consumption) is fixed (a point in Supplementary Figure. 4a). Therefore, theoretically, the best working point of ANNs is at the turning point $T_0 = \sum_{l=1}^{L} T_l^{ANN}$ (the red point in Supplementary Figure. 4a). The reason is that if ANNs work in the saturation region, such as the yellow point in Supplementary Figure. 4a, lower-power or cheaper chips can be used to achieve the same performance. Otherwise, in the undersaturation region, such as the grey point, some sampled data are wasted. In this case, sensors with high performance are not required.

For SNNs, owing to the event-driven information processing ability and local memory, SNNs can execute computations without the need to get the entire information ready. If SNNs input with an event or analogue sensor, the total time consumption yields

$$T_{SNNs} = \sum_{l=1}^{L} T_l^{SNN} = \sum_{l=1}^{L}\left(\frac{C_l^{SNN}}{\alpha} + \frac{M_l^{SNN}}{\beta}\right) \quad if\ T_{SNNs} > \tau. \tag{19}$$

where the τ indicates the refractory period of the input sensor or SNNs neurons (generally in DVS $\tau = 15\mu s$). Unlike ANNs, the T_l^{SNN} is related to the input, because different stimuli patterns will have a different output firing rate R_l. Therefore, as shown in Supplementary Figure. 4a, the time consumption of SNNs is adaptive between $\sum_{l=1}^{L} R_l /L = 1$ and $\sum_{l=1}^{L} R_l /L = 0$. Due to the limited information capacity in one time-step, most SNNs have a time window N to accumulate the final output. Typically, according to[30], SNNs require 20-512 or longer time steps to handle object tracking tasks. As shown in Supplementary Figure. 4b, the time consumption will be $W * T_{SNNs}$.

For HNNs, we propose a divide-and-conquer strategy, which processes the stationary (low-frequency) information by ANNs and transient (high-frequency) information by SNNs. The different network models work in a parallel mode, meaning that the SNN part of HNNs can produce an output at an arbitrary time without the accumulation of simulation time window. Assuming that the ANNs-part and SNNs-part

equally allocate computing resources ($\alpha_{max}/2$ and $\beta/2$ for each model) in the computation core, the time consumption of HNNs follows

$$T_{HNNs} = min[max(T_0, \sum_{l=1}^{L}(\frac{C_l^{ANN}}{\alpha} + \frac{M_l^{ANN}}{\beta})), \sum_{l=1}^{L}(\frac{C_l^{SNN}}{\alpha} + \frac{M_l^{SNN}}{\beta})]. \quad (20)$$

If the structures of the ANN part and SNN part in HNNs are the same, in most cases, the T_{HNNs} will be $\sum_{l=1}^{L}(\frac{C_l^{SNN}}{\alpha} + \frac{M_l^{SNN}}{\beta})$. Additionally, equally dividing computing resources is the worst but most straightforward strategy, which is common in GPUs. However, in FPGA or neuromorphic chips, we can design a dynamic resource allocation strategy to make $T_{HNNs} \approx T_{SNNs}$. As shown in Supplementary Figure. 4c, the T_{HNNs} follows $T_{SNNs} < T_{HNNs} < 2T_{SNNs}$.

In brief, HNNs inherit the event-driven and sparsity features of SNNs and can achieve a faster speed than ANNs and multiple time-step SNNs in the same task and hardware. Since the involvement of ANNs improves the single time-step representation capability, SNNs can work without a particular time window.

Supplementary References

1. Gallego G*, et al.* Event-based vision: A survey. *arXiv preprint arXiv:190408405*, (2019).
2. Liu S-C, Delbruck T. Neuromorphic sensory systems. *Current opinion in neurobiology* **20**, 288-295 (2010).
3. Brandli C, Berner R, Yang M, Liu S-C, Delbruck T. A 240× 180 130 db 3 µs latency global shutter spatiotemporal vision sensor. *IEEE Journal of Solid-State Circuits* **49**, 2333-2341 (2014).
4. Kiani Galoogahi H, Fagg A, Huang C, Ramanan D, Lucey S. Need for speed: A benchmark for higher frame rate object tracking. In: *Proceedings of the IEEE International Conference on Computer Vision*) (2017).
5. Kristan M*, et al.* The visual object tracking vot2017 challenge results. In: *Proceedings of the IEEE international conference on computer vision workshops*) (2017).
6. Niklaus S, Mai L, Liu F. Video frame interpolation via adaptive separable convolution. In: *Proceedings of the IEEE International Conference on Computer Vision*) (2017).
7. Li M, Wang Y-X, Ramanan D. Towards Streaming Perception. In: *European Conference on Computer Vision*). Springer (2020).
8. Huang Z, Zhan J, Zhao H, Lin K, Zheng P, Lv J. Real-Time Visual Tracking Base on SiamRPN with Generalized Intersection over Union. In: *International Conference on Brain Inspired Cognitive Systems*). Springer (2019).
9. Hornik K. Approximation capabilities of multilayer feedforward networks. *Neural Networks* **4**, 251-257 (1991).
10. Blum EK, Li LK. Approximation theory and feedforward networks. *Neural Networks* **4**, 511-515 (1991).
11. Kůrková V. Kolmogorov's theorem and multilayer neural networks. *Neural Networks* **5**, 501-506 (1992).

12. Maass W, Natschläger T, Markram H. Real-time computing without stable states: A new framework for neural computation based on perturbations. *Neural computation* **14**, 2531-2560 (2002).
13. Maass W, Markram H. On the computational power of circuits of spiking neurons. *Journal of computer and system sciences* **69**, 593-616 (2004).
14. Maass W. Networks of spiking neurons: The third generation of neural network models. *Neural Networks* **10**, 1659-1671 (1997).
15. Hendry SH, Reid RC. The koniocellular pathway in primate vision. *Annual review of neuroscience* **23**, 127-153 (2000).
16. Silveira LCL*, et al.* Morphology and physiology of primate M-and P-cells. *Progress in brain research* **144**, 21-46 (2004).
17. Stein J. Dyslexia: the role of vision and visual attention. *Current developmental disorders reports* **1**, 267-280 (2014).
18. Callaway EM. Structure and function of parallel pathways in the primate early visual system. *The Journal of physiology* **566**, 13-19 (2005).
19. Abbott L, Regehr WG. Synaptic computation. *Nature* **431**, 796-803 (2004).
20. Bargmann CI. Beyond the connectome: how neuromodulators shape neural circuits. *Bioessays* **34**, 458-465 (2012).
21. Bucher D, Marder E. SnapShot: neuromodulation. *Cell* **155**, 482-482. e481 (2013).
22. Smith SJ*, et al.* Single-cell transcriptomic evidence for dense intracortical neuropeptide networks. *Elife* **8**, e47889 (2019).
23. Smith SJ, Hawrylycz M, Rossier J, Sümbül U. New light on cortical neuropeptides and synaptic network plasticity. *Current Opinion in Neurobiology* **63**, 176-188 (2020).
24. Greff K, van Steenkiste S, Schmidhuber J. On the Binding Problem in Artificial Neural Networks. *arXiv preprint arXiv:201205208*, (2020).
25. Papadimitriou CH, Vempala SS, Mitropolsky D, Collins M, Maass W. Brain computation by assemblies of neurons. *Proceedings of the National Academy of Sciences* **117**, 14464-14472 (2020).
26. György Buzsáki M. *The brain from inside out*. Oxford University Press (2019).
27. Donald EK. The art of computer programming. *Sorting and searching* **3**, 426-458 (1999).
28. Williams S, Waterman A, Patterson D. Roofline: an insightful visual performance model for multicore architectures. *Communications of the ACM* **52**, 65-76 (2009).
29. Choquette J, Gandhi W, Giroux O, Stam N, Krashinsky R. Nvidia a100 tensor core gpu: Performance and innovation. *IEEE Micro* **41**, 29-35 (2021).
30. Luo Y*, et al.* SiamSNN: Spike-based Siamese Network for Energy-Efficient and Real-time Object Tracking. *arXiv preprint arXiv:200307584*, (2020).
